# Mechanisms and Role of Dendritic Membrane Trafficking for Long-Term Potentiation

**DOI:** 10.3389/fncel.2018.00391

**Published:** 2018-10-30

**Authors:** Brian G. Hiester, Matthew I. Becker, Aaron B. Bowen, Samantha L. Schwartz, Matthew J. Kennedy

**Affiliations:** ^1^Department of Pharmacology, University of Colorado School of Medicine, Aurora, CO, United States; ^2^Department of Physiology and Biophysics, University of Colorado School of Medicine, Aurora, CO, United States

**Keywords:** long term potentiation, AMPA receptor, exocytosis, dendrite, membrane trafficking, recycling endosomes, dendritic spines, plasticity and learning

## Abstract

Long-term potentiation (LTP) of excitatory synapses is a major form of plasticity for learning and memory in the central nervous system. While the molecular mechanisms of LTP have been debated for decades, there is consensus that LTP induction activates membrane trafficking pathways within dendrites that are essential for synapse growth and strengthening. Current models suggest that key molecules for synaptic potentiation are sequestered within intracellular organelles, which are mobilized by synaptic activity to fuse with the plasma membrane following LTP induction. While the identity of the factors mobilized to the plasma membrane during LTP remain obscure, the field has narrowly focused on AMPA-type glutamate receptors. Here, we review recent literature and present new experimental data from our lab investigating whether AMPA receptors trafficked from intracellular organelles directly contribute to synaptic strengthening during LTP. We propose a modified model where membrane trafficking delivers distinct factors that are required to maintain synapse growth and AMPA receptor incorporation following LTP. Finally, we pose several fundamental questions that may guide further inquiry into the role of membrane trafficking for synaptic plasticity.

## Introduction

Information storage, learning, and adaptive behavior are thought to occur through use-dependent changes in the strength of synaptic connections. For example, long-term potentiation (LTP) of excitatory synapses is widely accepted as a critical form of plasticity for learning and memory throughout the brain (Bliss and Collingridge, 1993; Malenka and Nicoll, 1999; Nicoll, 2017). While numerous pre- and postsynaptic LTP mechanisms have been described in diverse circuits, LTP has been most intensely investigated in pyramidal neurons of hippocampal region CA1. Here, multiple lines of evidence agree that LTP is predominantly mediated by increased function of postsynaptic α-amino-3-hydroxy-5-methyl-4-isoxazolepropionic acid (AMPA)-type glutamate receptors. Increased channel conductance, open probability, and receptor number have all been reported to be responsible for synaptic potentiation (Isaac et al., 1995; Liao et al., 1995; Roche et al., 1996; Barria et al., 1997; Benke et al., 1998; Derkach et al., 1999; Shi et al., 1999; Banke et al., 2000). Support for increased number of synaptic AMPA receptors during LTP primarily comes from biochemical measurements demonstrating the level of surface receptors increases following LTP and from microscopy experiments directly visualizing tagged AMPA receptors as they cluster at postsynaptic sites following LTP induction (Shi et al., 1999; Heynen et al., 2000; Broutman and Baudry, 2001; Lu et al., 2001). Functional studies using peak-scaled non-stationary fluctuation analysis to estimate changes in receptor number and conductance following LTP are also consistent with insertion of AMPA receptors into the postsynaptic density (PSD) during LTP (Benke and Traynelis, 2018). While there is general agreement that AMPA receptors are recruited to the postsynaptic plasma membrane (PM) following LTP, the source of these receptors remains controversial. Two major pools of “extrasynaptic” receptors are available: those that are already laterally diffusing within the dendritic PM, and those that are housed in internal membrane-bound organelles. Thus, AMPA receptors could be added to the postsynaptic membrane by trapping diffusing surface receptors and/or through mobilizing receptors from internal stores. The latter mechanism requires that intracellular organelles housing AMPA receptors fuse near the postsynaptic membrane to deliver receptors to synapses undergoing plasticity. The early observation that LTP depends on membrane fusion provides tantalizing support for mobilization of receptors from intracellular pools. While there is strong evidence that AMPA receptors are mobilized to the PM during LTP, no study has definitively demonstrated this pool of receptors directly contributes to synapse potentiation. On the contrary, recent experiments support a major role for trapping laterally diffusing receptors at synaptic sites during LTP. Here we discuss literature supporting both sides of this issue and provide experimental data from our lab consistent with a model where membrane fusion delivers as-yet unidentified factors that stabilize AMPA receptors at synaptic sites following their initial incorporation by lateral diffusion.

### Membrane trafficking is essential for LTP

While the molecular mechanisms that govern LTP have been debated for decades, there is general consensus that membrane trafficking in the postsynaptic cell is essential. This was first reported by Lledo et al. (1998) who demonstrated that infusing postsynaptic neurons with factors that inhibit membrane fusion mediated by soluble N-ethylmaleimide sensitive factor attachment protein receptor (SNARE) family proteins, including a peptide that disrupts SNAP interactions, N-ethylmaleimide and botulinum neurotoxin B, blocked LTP. Intriguingly, none of these reagents affected the initial magnitude of synaptic potentiation that occurred following LTP induction, which likely arises from post-tetanic potentiation of neurotransmitter release and enhanced postsynaptic AMPA receptor function and/or number. However, synaptic responses gradually declined to baseline levels ~20–30 min following LTP induction when membrane fusion was disrupted. These experiments provided the first evidence that membrane fusion in the postsynaptic cell is required for sustained synaptic potentiation during LTP. Given that the initial phase of LTP appeared normal when membrane fusion was blocked, these experiments also demonstrate that the trafficking requirement does not manifest until several minutes following LTP induction. Numerous subsequent studies using diverse LTP induction protocols and recording techniques have established postsynaptic membrane trafficking as a hallmark of LTP (Lu et al., 2001; Park et al., 2004, 2006; Kopec et al., 2007; Yang et al., 2008b).

Given the central importance of postsynaptic membrane fusion for LTP, a critical question is the identity of the organelle(s) undergoing fusion. There is a vast network of intracellular organelles present within neuronal dendrites and spines (Parton et al., 1992; Spacek and Harris, 1997; Cooney et al., 2002; Park et al., 2004, 2006; Rácz et al., 2004; Kennedy et al., 2010; Hanus et al., 2014; Esteves da Silva et al., 2015; Bowen et al., 2017; Hiester et al., 2017; Wu et al., 2017b). Among the organelles that could participate in rapid membrane remodeling at synapses, recycling endosomes (REs) stand out. REs are intracellular vesicles that regulate trafficking of protein cargoes to and from the PM (Maxfield and McGraw, 2004). In neurons, REs are distributed throughout the dendritic arbor and within a substantial fraction of dendritic spines. Importantly, REs are mobilized to fuse with the PM following LTP stimuli, resulting in the rapid delivery of resident RE cargo proteins to the dendritic surface (Park et al., 2006; Wang et al., 2008c; Kennedy et al., 2010; Keith et al., 2012; Roman-Vendrell et al., 2014; Woolfrey et al., 2015; Hiester et al., 2017). Importantly, disruption of postsynaptic RE function also disrupts functional LTP and accompanying morphological plasticity (Park et al., 2004, 2006; Brown et al., 2007; Wang et al., 2008c; Kennedy et al., 2010; Keith et al., 2012; Woolfrey et al., 2015). Activity triggered RE fusion occurs throughout neuronal dendrites, including within dendritic spine heads suggesting that the excitatory postsynaptic membrane could be rapidly remodeled via nearby RE fusion, although the precise location (i.e., spine head vs. dendritic shaft) of the RE fusion events relevant for LTP remains a controversial and open question (Spacek and Harris, 1997; Cooney et al., 2002; Rácz et al., 2004; Yudowski et al., 2007; Lin et al., 2009; Makino and Malinow, 2009; Kennedy et al., 2010; Patterson et al., 2010; Hiester et al., 2017; Wu et al., 2017b). Regardless of whether the LTP-relevant RE fusion events occur within or near activated spines, REs must be able to sense local activity in order to fuse near synapses undergoing LTP. While few studies have investigated the spatial relationship between activated synapses and RE fusion, Patterson et al., demonstrated that glutamate uncaging over individual dendritic spines triggers fusion of GluA1-containing vesicles both within the activated spine and in the nearby dendritic shaft (Patterson et al., 2010). This finding was supported by a subsequent study demonstrating that glutamate uncaging triggers RE fusion within activated spines, consistent with a role for RE fusion in synapse-specific GluA1 delivery events observed by Patterson et al. (Hiester et al., 2017). Whether spine RE fusion plays a direct role in LTP remains an open question, but at steady state, not all dendritic spines house REs raising the issue of whether spines lacking a resident RE are impaired for LTP. Intriguingly, LTP measured by long-lasting morphological spine growth following single spine glutamate uncaging was originally reported to occur in 55% of spines (and less frequently at larger spines) and functional LTP at presumed single synapses occurred at 65% of synapses tested, similar to the fraction of RE-containing spines, which has been reported at 25–50% depending on age and endosome classification criteria (Petersen et al., 1998; Cooney et al., 2002; Matsuzaki et al., 2004; Park et al., 2006; Kennedy et al., 2010; Hiester et al., 2017). More refined local RE inactivation techniques will be required to begin addressing the spatial relationship between RE fusion and synapses undergoing LTP.

### AMPA receptors localize to dendritic REs and are mobilized to the cell surface by synaptic activity

Given that membrane fusion and RE function is essential for LTP, a central issue is the identity of the cargo delivered to synapses via RE fusion events. Because synaptic AMPA receptor content increases following LTP, many studies focused on determining whether RE fusion could be the major delivery route to the synapse. Indeed, an immunoelectron microscopy-based investigation of the ultrastructural localization of internal AMPA receptors identified a population of GluA2 that localizes to dendritic, but not spine endosomes, although peri-synaptic endocytic pits could be observed to contain GluA2 following NMDA receptor activation (Tao-Cheng et al., 2011). Using a sensitive antibody feeding assay to selectively label internalized pools of AMPA receptors, multiple studies have demonstrated localization of AMPA receptor subunits GluA1 and GluA2 to a large fraction of REs within dendritic shafts and spines (Ehlers, 2000; Park et al., 2004; Kennedy et al., 2010; Hiester et al., 2017). In these studies, the majority of internalized GluA1 co-localizes with RE marker proteins, supporting a major role for these organelles in AMPA receptor surface trafficking, though it is possible that constitutive trafficking of AMPA receptors occurs through a subset of REs, positive for the small GTPases Arf6 and TC10 (Zheng et al., 2015). Indeed, the molecular and functional heterogeneity of endosomes labeled with classical markers such as transferrin receptor or rab proteins deserves further investigation. For example, it remains unknown what mechanisms allow a subset of endosomes to be mobilized by synaptic activity to fuse with the PM. More direct support for regulated AMPA receptor surface delivery via REs came from experiments using an NMDA receptor-dependent chemical LTP (cLTP) stimulation. Because this form of stimulation globally activates many synaptic inputs, potentiation can be monitored by measuring the amplitude and frequency of spontaneous miniature excitatory postsynaptic currents (mEPSCs) mediated by AMPA receptors and correlated with surface GluA1 levels measured by immunolabeling (Lu et al., 2001; Park et al., 2004). Following cLTP, surface GluA1 was elevated and mEPSC amplitude increased, providing a positive correlation between synapse potentiation and GluA1 delivery to the PM (Lu et al., 2001). Importantly, both potentiated mEPSC amplitude and increased surface GluA1 were blocked by tetanus neurotoxin (TeNT), which cleaves the vesicle associated membrane proteins (VAMPs) required for activity-dependent membrane fusion in axons and dendrites (Maletic-Savatic and Malinow, 1998; Lu et al., 2001). Subsequent studies utilizing similar cLTP stimuli also demonstrated that surface levels of endogenous (Ahmad et al., 2012; Jaafari et al., 2012, 2013; Jurado et al., 2013; Hiester et al., 2017; Wu et al., 2017a) and exogenously expressed (Passafaro et al., 2001; Park et al., 2004; Patterson et al., 2010) GluA1-containing AMPA receptors increase following stimulation. Many of these studies additionally demonstrated that the same SNARE machinery that is required for expression of LTP is also required for AMPA receptor surface delivery (Ahmad et al., 2012; Jurado et al., 2013; Wu et al., 2017a; Bin et al., 2018). Importantly, disrupting RE function also blocks regulated AMPA receptor surface delivery, synapse potentiation, and spine growth following LTP stimuli, supporting a model where REs are the primary organelles undergoing fusion for excitatory synaptic plasticity (Park et al., 2004, 2006; Brown et al., 2007; Wang et al., 2008c; Kennedy et al., 2010).

**Figure 1:**
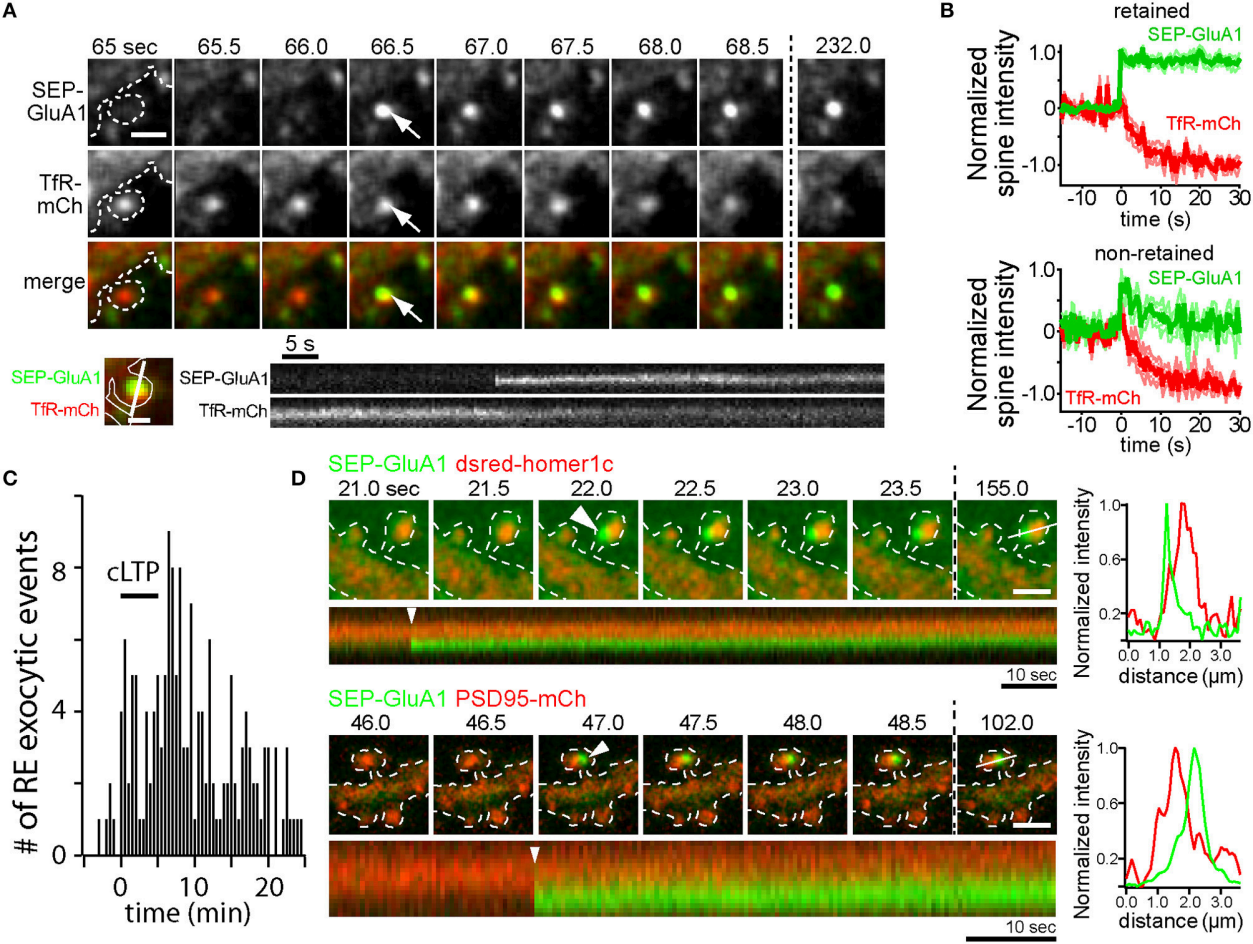
AMPA receptors can be directly inserted into the spine plasma membrane adjacent to the PSD by RE fusion. **(A)** Time-lapse imaging of a hippocampal neuron coexpressing SEP-GluA1 (top row) and the RE marker protein TfR-mCh (middle row) following cLTP stimulation. Note the abrupt appearance of SEP- GluA1 fluorescence at the precise location of a spine RE (arrows). Scale bar, 1 μm. Below is a kymograph showing a single spine exocytic event of SEP-GluA1 (top) and TfR-mCh (bottom). Note that SEP-GluA1 is inserted and retained in the spine head, even as TfR-mCh from the same fusion event quickly diffuses away. Scale bar, 0.5 μm. **(B)** SEP-GluA1 exocytic events fall into two classes. Following RE fusion, SEP-GluA1 was either retained in spines (62% of total events, top panel) or quickly diffused away (38% of total events, bottom panel). In all cases, colocalized TfR-mCh signal (red traces) declined rapidly following the appearance of SEP-GluA1 (green traces). To average traces from multiple events, individual traces were aligned at the time of fusion, which was arbitrarily labeled *t* = 0 s. **(C)** The timing of RE fusion events within dendritic spines before, during and following cLTP stimulus (black bar) is plotted. Data in panels **(A–C)** were modified from Kennedy et al. (2010) and reprinted with permission from *Cell Press*. **(D)** AMPA receptors are inserted adjacent to the PSD. Time lapse imaging of hippocampal neurons coexpressing SEP-GluA1 and dsred-homer1c (top) or PSD95-mCh (bottom) following cLTP stimulation. Discrete SEP-GluA1 insertion events (arrowheads) occurred adjacent to, but not directly overlapping, the PSD. Scale bar, 1 μm. A kymograph measured along the line from the final time point is shown below each time series, and an intensity profile of the signal from each channel at the time of exocytosis (*t* = 0 s, arrowhead) is shown on the right demonstrating the two signals are optically resolvable.

A complementary line of inquiry utilized longitudinal live-cell microscopy to directly visualize AMPA receptor trafficking during LTP. One of the most widely used techniques relies upon the pH sensitive green fluorescent protein superecliptic pHluorin (SEP), which is brightly fluorescent at neutral pH, but quenched within the acidic lumen of intracellular endosomes (Miesenbock et al., 1998). Numerous studies have used SEP-GluA1 to monitor activity-triggered AMPA receptor membrane insertion (Kopec et al., 2007; Yudowski et al., 2007; Makino and Malinow, 2009; Petrini et al., 2009; Kennedy et al., 2010; Patterson et al., 2010). Following global cLTP stimulation, the frequency of SEP-GluA1 insertion events increases, indicating that internal stores of GluA1 are mobilized to the dendritic PM in an NMDA receptor-dependent manner (Yudowski et al., 2007). Further, activity-triggered SEP-GluA1 insertion events are inhibited by botulinum neurotoxins A and TeNT which cleave SNAP25 and VAMP family proteins respectively, providing another correlative link between functional LTP and AMPA receptor delivery to the PM (Makino and Malinow, 2009; Patterson et al., 2010). Similar global stimulation approaches and more refined single synapse glutamate uncaging techniques induce SEP-GluA1 insertion directly within dendritic spines (Kennedy et al., 2010; Patterson et al., 2010). Direct spine SEP-GluA1 delivery is the result of RE fusion, as demonstrated by dual color imaging of a RE marker protein along with SEP-GluA1 (Kennedy et al., 2010) (Figures 1A,B). Intriguingly, the timing of spine RE fusion is highly variable following the onset of stimulation. Figure 1C shows the timing of spine RE fusion before, during and following cLTP stimulation. While RE fusion can occur immediately following stimulation, many events in spines (and dendritic shafts) occur several minutes following stimulation (Kennedy et al., 2010). The broad timing of the events relative to the onset of stimulation raises the intriguing possibility that different subtypes of endosomes can differentially respond to activity to deliver distinct cargoes during different phases of plasticity. Alternatively, global stimulation paradigms where many synapses are simultaneously activated could deplete resources required for membrane fusion and therefore influence when and where the events occur. Indeed, the timing of spine RE fusion events was more tightly correlated with the onset of stimulation when individual synapses were activated using glutamate uncaging, but could still occur tens of seconds to minutes following stimulation (Patterson et al., 2010; Hiester et al., 2017).

Collectively, these studies point toward a mechanism whereby NMDA receptor activation during LTP drives Ca^2+^-dependent fusion of intracellular REs, thus delivering GluA1-containing AMPA receptors to the cell surface. However, none of these studies demonstrate that newly delivered receptors play a direct role in potentiating synaptic responses. For example, the extent to which AMPA receptors recently trafficked to the cell surface stably incorporate into dendritic spines remains controversial with some studies demonstrating that SEP-GluA1 inserted into the dendritic shaft transiently enters spines but is not trapped (Yudowski et al., 2007; Makino and Malinow, 2009) and others demonstrating some degree of receptor trapping following direct insertion into spines (Kennedy et al., 2010; Patterson et al., 2010) (Figures 1A,B). In many of these studies SEP-GluA1 insertion events were relatively rare. For example, Patterson et al. demonstrate that newly inserted receptors contribute only 10–30% of the total accumulated spine SEP-GluA1 fluorescence following LTP induced by glutamate uncaging (Patterson et al., 2010). However, it should be noted that SEP-GluA1 experiments should be interpreted with caution. Data from our lab has shown that SEP-GluA1 localization to REs is substantially lower than that observed using more sensitive antibody feeding techniques to selectively quantify internal pools of endogenous GluA1 and GluA2 (Kennedy et al., 2010; Hiester et al., 2017). The reason for this is unclear, but multiple studies have demonstrated that under basal conditions, N-terminally tagged GluA1 receptors do not efficiently integrate into synaptic sites (Díaz-Alonso et al., 2017; Watson et al., 2017). Thus, decreased recycling pools of SEP-GluA1 could arise from lack of agonist-induced internalization since they may not be activated under basal conditions. In any case, given the sparseness of endosomal SEP-GluA1, this approach likely underestimates the fraction of newly inserted endogenous receptors during LTP, making it difficult to determine when, where and whether newly inserted receptors could directly contribute to the LTP response. Furthermore, spine localization observed with traditional confocal microscopy does not necessarily prove that receptors contribute to synaptic function. For example, recent work from our lab and others have demonstrated that receptors in and adjacent to the PSD may not be functionally activated unless they are precisely positioned within sub-PSD nanodomains directly opposite sites of neurotransmitter release (MacGillavry et al., 2013; Tang et al., 2016; Biederer et al., 2017; Sinnen et al., 2017; Hruska et al., 2018). Indeed, we present new imaging experiments simultaneously visualizing PSD markers along with SEP-GluA1 spine insertion events. These events were rare due to the sparseness of detectable endosomal SEP-GluA1, but when they occured SEP-GluA1 remained optically resolvable from the PSD for at least several minutes following insertion (Figure 1D). While this observation demonstrates perisynaptic fusion of SEP-GluA1-containing endosomes, the fact that newly inserted receptors remain resolvable from the PSD should be interpreted with caution since movement into the PSD could be hindered by the N-terminal SEP tag through steric interference and/or disruption of N-terminal binding interactions (Díaz-Alonso et al., 2017; Watson et al., 2017). While SEP-GluA1 can be retained in perisynaptic regions within spines following membrane insertion, co-trafficking TfR-mCh reaching the surface in the same fusion event rapidly diffuses from the site of insertion, demonstrating a selective trapping mechanism for AMPA receptors (Figures 1A,B,D) (Kennedy et al., 2010). The molecular mechanisms responsible for spine trapping and the extent to which native receptors integrate into the PSD following surface delivery will require new approaches for labeling and tracking endogenous receptors (Wakayama et al., 2017).

### Assessing the role of lateral diffusion vs. membrane trafficking for AMPA receptor delivery during LTP

While it is generally agreed that diverse LTP stimuli trigger AMPA receptor delivery to the cell surface, whether newly delivered receptors directly contribute to the LTP response remains a fundamental question. Alternatively, fast lateral diffusion and trapping of receptors already present at the surface may be the primary driver of increased synaptic AMPA receptor number during LTP. Indeed, a pool of AMPA receptors laterally diffuses in the PM, where they frequently encounter synaptic sites (Borgdorff and Choquet, 2002; Bats et al., 2007; Ehlers et al., 2007; Petrini et al., 2009; Opazo et al., 2010). Given their fast activation and desensitization kinetics, a rapidly exchanging pool of receptors is thought to be required to sustain high-frequency neurotransmission (Heine et al., 2008a). Intriguingly, AMPA receptor surface diffusion is regulated by synaptic activity, which generally increases mobility (Tardin et al., 2003; Groc et al., 2004). For example Groc et al. (2004) demonstrate that neural stimulation increases mobility of extrasynaptic receptors, largely through liberating a pool of immobile receptors. This could result in an expanded pool of diffusing receptors for synaptic integration and potentiation. Diffusing AMPA receptors can be trapped at synaptic sites through interactions between transmembrane AMPA receptor regulatory proteins (TARPs) and synaptic scaffolding proteins (Borgdorff and Choquet, 2002; Ashby et al., 2006; Makino and Malinow, 2009). Accumulation of laterally diffusing AMPA receptors is regulated by synaptic activity (Ehlers et al., 2007; Makino and Malinow, 2009; Petrini et al., 2009) in a manner that requires CaMKII phosphorylation of TARPs to promote anchoring of receptors to the postsynaptic scaffold protein PSD-95 (Hayashi et al., 2000; Schnell et al., 2002; Bats et al., 2007; Opazo et al., 2010). Activity-triggered trapping of laterally diffusing AMPA receptors occurs on rapid time scales (<1 min) (Petrini et al., 2009; Opazo et al., 2010), consistent with early synaptic potentiation that occurs within seconds to minutes following LTP induction. Thus, at least one mechanism has been described that could account for the rapid incorporation of extrasynaptic surface AMPA receptors into the postsynaptic membrane without a requirement for membrane trafficking.

**Figure 2:**
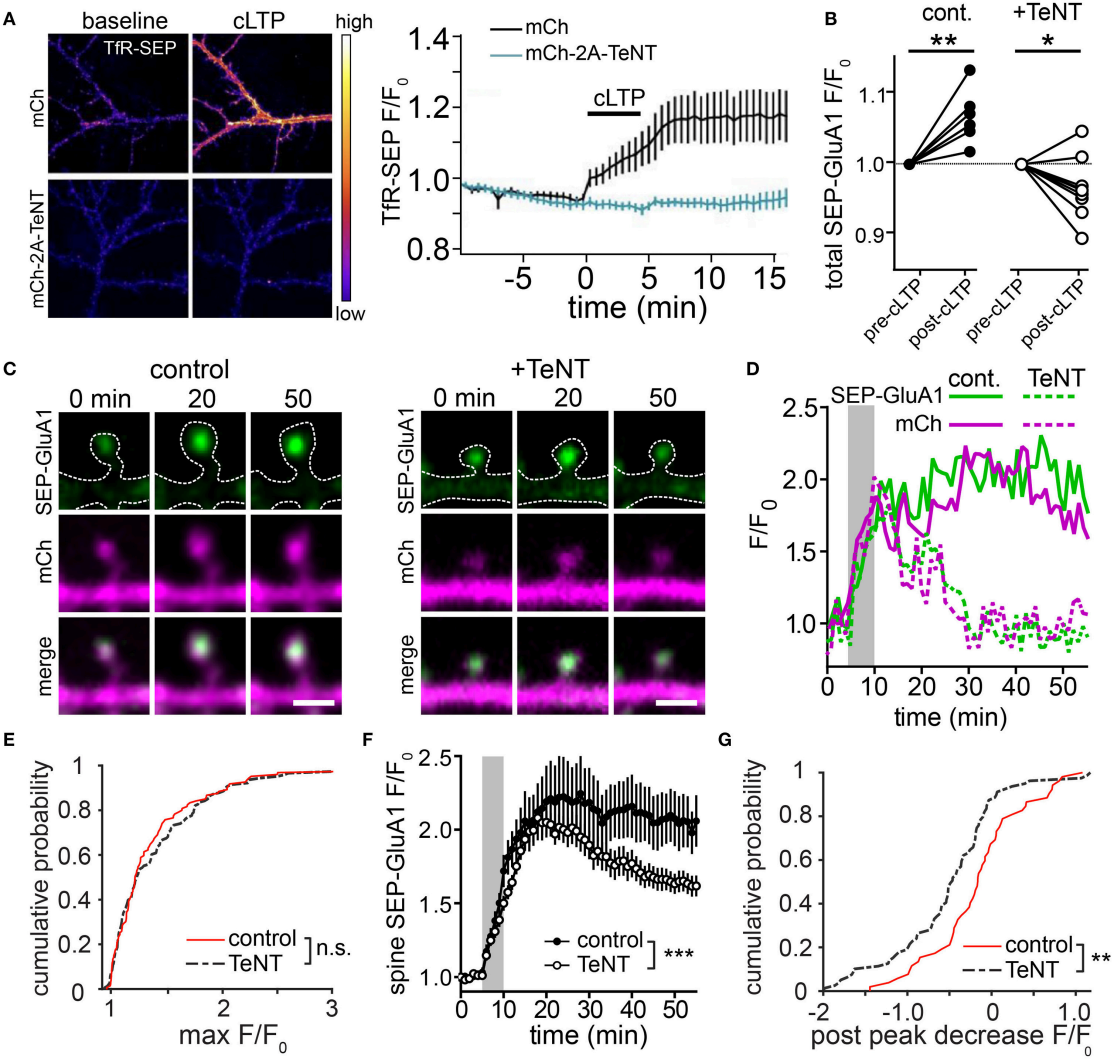
Blocking postsynaptic exocytosis prevents stabilization of AMPA receptors initially recruited by activity in dendritic spines. **(A)** TeNT light chain blocks activity-triggered RE fusion and accumulation of surface TfR-SEP. Representative images of dissociated hippocampal neurons expressing TfR-SEP with mCherry (mCh, top panels) or mCh and TeNT (mCh-2A-TeNT, bottom panels). Images were taken pre- (left) and 15 min post (right) cLTP stimulation. The plot to the right shows TfR-SEP signal plotted as a function of time following cLTP (black bar). *N* = 8–10 neurons per condition. **(B)** Quantification of the normalized total dendritic SEP-GluA1 signal before and after cLTP stimulation for control (*n* = 7 neurons) and TeNT expressing (*n* = 9 neurons) neurons. ^**^p < 0.001, ^*^p < 0.05 (Paired two-tailed Student's *t*-test). **(C)** Representative examples of SEP-GluA1 spine accumulation from control (left) and TeNT expressing (right) neurons before (0 min), 20 and 50 min following cLTP stimulation. The top row shows the SEP-GluA1 signal, the middle row shows the mCh signal and the bottom row shows the merge of the two channels. The dotted line represents an outline of the cell morphology based on the mCh signal. Scale bar, 1 μm. **(D)** Traces showing the spine SEP-GluA1 and mCh signals in control (solid lines) and TeNT-expressing (dashed lines) neurons as a function of time for the spines shown in **(C)**. The gray box indicates the duration of the cLTP stimulus. **(E)** Cumulative probability of maximum SEP-GluA1 accumulation within randomly selected spines following cLTP stimulation for control neurons (red line, *n* = 119 spines from 6 neurons) and neurons expressing TeNT (black dashed line, *n* = 138 spines from 9 neurons). *p* = 0.66, Kolmogorov-Smirnov test. **(F)** Quantification of the SEP-GluA1 signal retention over time in selected dendritic spines for control (filled circles, *n* = 52 spines from 7 neurons) and TeNT expressing (open circles, *n* = 87 spines from 9 neurons) neurons. Only spines that acquired SEP signal >25% over baseline were selected for this analysis. The gray box indicates the duration of the cLTP stimulus. ^***^p < 0.001 (Two-way ANOVA, Bonferroni multiple comparisons test). **(G)** Shown is a cumulative probability plot of the decrease in spine SEP-GluA1 signal 50 min following cLTP stimulation in control neurons (red line) and neurons expressing TeNT (black dashed line). *p* = 0.0082, Kolmogorov-Smirnov test.

**Figure 3:**
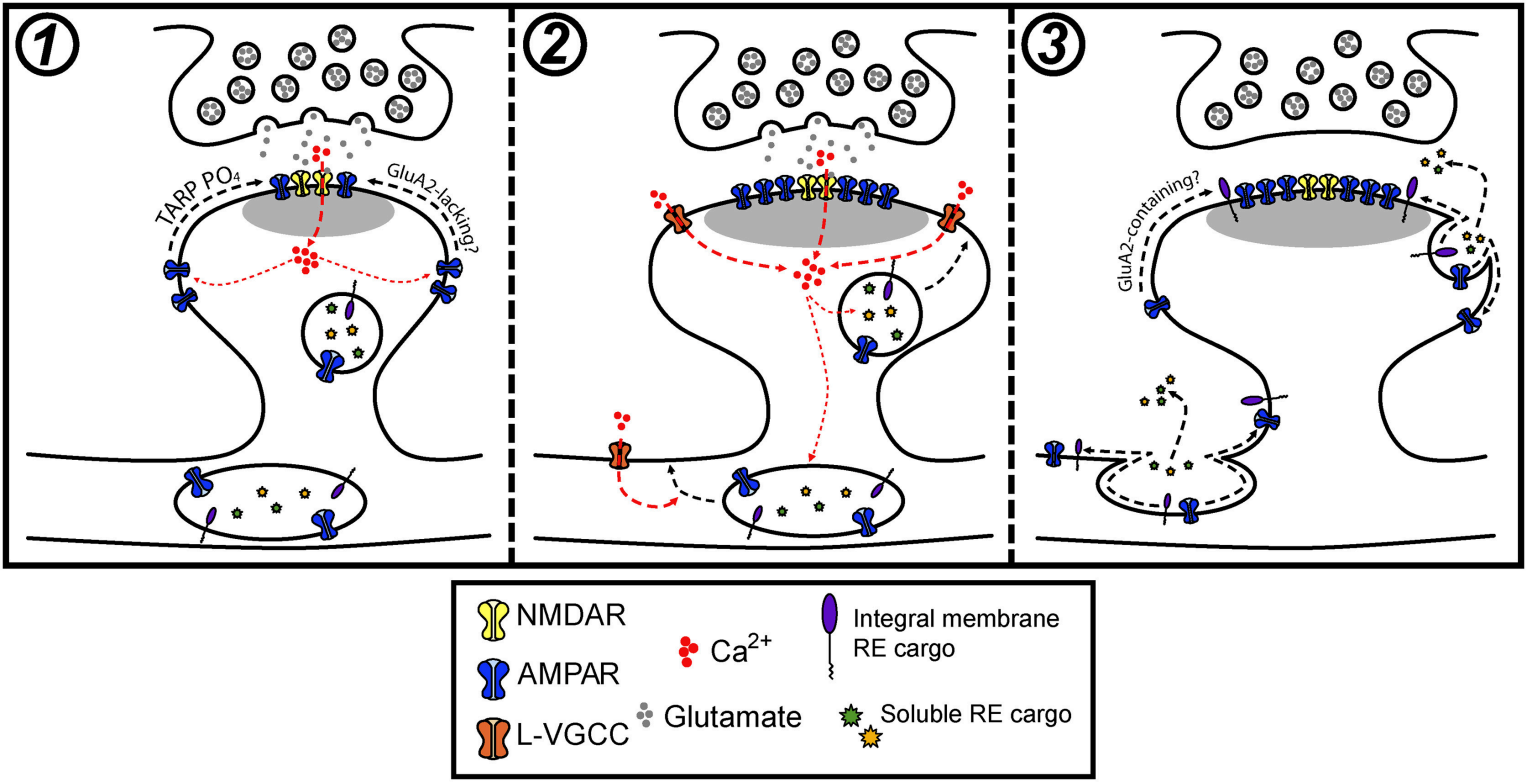
Model for how postsynaptic membrane trafficking regulates AMPA receptor accumulation and stabilization during LTP. **(1)** The onset of an LTP stimulus promotes Ca^2+^ entry through NMDA receptors, which drives the initial trapping of pre-existing surface diffusing AMPA receptors at synapses. This can occur through activity-triggered phosphorylation of TARPs and subsequent binding to PSD scaffolds. **(2)** NMDA receptor and L-VGCC activation cooperatively promote the fusion of intracellular vesicles in dendrites and spines with the PM. **(3)** Activity-triggered fusion of postsynaptic vesicles delivers key synaptic cargoes, including AMPA receptors and other integral membrane proteins along with secreted soluble proteins to the PM. The delivery of one or a combination of these factors contributes to the stabilization of synaptic AMPA receptors that accumulated during the early stages of LTP.

To more directly assess the role of lateral diffusion vs. membrane trafficking for synaptic delivery of AMPA receptors, a recent study employed an acute crosslinking approach to prevent lateral diffusion of surface AMPA receptors prior to LTP induction (Penn et al., 2017). In this study, either neutravidin crosslinking of expressed, biotinylated GluA2-containing AMPA receptors, or antibody crosslinking of endogenous GluA2 subunits blocked the earliest phase of LTP that occurs within seconds to minutes following induction. This observation supports a model where the rapid, initial phase of synapse potentiation is driven by lateral diffusion of GluA2-containing receptors into the postsynaptic membrane. Interestingly, when receptors were crosslinked prior to LTP induction, postsynaptic responses slowly increased for tens of minutes following LTP induction. This gradual potentiation was blocked by TeNT, consistent with slow synaptic accumulation of newly inserted receptors that were not subject to pre-induction crosslinking. Importantly, the magnitude of the slow increase in synaptic responses was significantly smaller than the control LTP response, suggesting that receptors newly trafficked to the cell surface play a relatively minor role in the LTP response. Finally, inclusion of neutravidin to crosslink GluA2-containing AMPA receptors during the entire timeframe of the experiments blocked both the rapid and gradual phases of synaptic potentiation indicating that receptors newly trafficked to the PM also must laterally diffuse into the postsynaptic membrane. This observation is consistent with dendritic and peri-synaptic fusion of REs, whose AMPA receptor cargo would need to laterally diffuse into the PSD to contribute to synaptic function (Figures 1C,D). It should be noted that it is also possible that newly inserted AMPA receptors (or GluA2-lacking receptors already present on the cell surface prior to LTP induction) could be blocked from entering functional domains within the PSD by pre-existing, crosslinked and immobilized GluA2-containing receptors. This interpretation could explain an apparent discrepancy between Penn et al. (2017), where GluA2-containing receptors were immobilized, and previous work demonstrating that GluA2-lacking receptors are initially responsible for synapse potentiation during initial stages (first ~25 min) of LTP (Plant et al., 2006). Nevertheless, the results from Penn et al. (2017) are also consistent with a major role for lateral diffusion in the initial synaptic potentiation that occurs following LTP, leading to a model where activity-triggered postsynaptic vesicle fusion promotes stability of AMPA receptors already recruited to synapses by lateral diffusion. We sought to further test this model using a complementary approach where we directly visualized AMPA receptors following synaptic stimulation when regulated membrane fusion was blocked with the catalytic light chain of tetanus neurotoxin (TeNT). To assess the efficiency of TeNT in blocking regulated dendritic membrane fusion, we co-expressed TeNT along with TfR-SEP in dissociated hippocampal neurons. Numerous previous studies have shown that cLTP stimulation triggers robust fusion of TfR-containing REs with the PM, resulting in an overall increase in surface TfR-SEP signal (Figure 2A) (Park et al., 2006; Kennedy et al., 2010). Activity-triggered surface insertion of TfR-SEP was completely blocked in TeNT-expressing neurons, confirming the efficacy of TeNT in blocking regulated RE fusion during cLTP. We next tested the effects of TeNT on AMPA receptor surface delivery and synapse accumulation following cLTP. As in previous studies, we imaged live neurons expressing the AMPA receptor subunit GluA1 tagged extracellularly with superecliptic pHluorin (SEP), which allowed us to quantitatively track surface accumulation and retention of surface AMPA receptors at individual spines following LTP induction (Ashby et al., 2006; Kopec et al., 2007; Yudowski et al., 2007; Makino and Malinow, 2009; Petrini et al., 2009; Kennedy et al., 2010; Patterson et al., 2010). In contrast to many previous studies, we imaged cells with minimal SEP-GluA1 expression levels where clear synaptic enrichment could be observed, resembling endogenous AMPA receptor distribution (and not simply an outline of the entire dendritic membrane). We measured total and spine-specific SEP-GluA1 signal during a 5 min baseline period and then exposed neurons to a cLTP stimulus. Quantification of total SEP signal was carried out several minutes following stimulation as others and we have observed rapid, stimulus dependent quenching of SEP-GluA1 signal specifically in the dendritic shaft during the cLTP stimulus, presumably due to the transient acidification of the endoplasmic reticulum that occurs upon NMDA receptor activation (Supplementary Figure 1) (Rathje et al., 2013). Under these conditions, we observed a modest, but significant elevation in total surface SEP-GluA1 following cLTP stimulation in control neurons (Figure 2B), consistent with previous studies (Petrini et al., 2009; Zhang et al., 2015). Interestingly, we observed a much more robust enrichment of SEP-GluA1 at dendritic spines (Figures 2C,D; Video 1). This increase mirrored spine growth measured with an mCh cell fill but is not simply a reflection of increased membrane surface area since we observed a robust enrichment of receptors within spines compared to the surrounding dendritic shaft (Figure 2C) (Lang et al., 2004; Matsuzaki et al., 2004; Kopec et al., 2006; Ehrlich et al., 2007; Yang et al., 2008a; Patterson et al., 2010). Increased spine SEP-GluA1 signal was frequently maintained for the duration of the imaging period (45 min post cLTP) (Figures 2D–G). To directly test the role of postsynaptic membrane fusion in contributing to spine GluA1 accumulation, we compared control neurons with neurons expressing the catalytic light chain of TeNT. While TeNT did not affect total basal surface levels of SEP-GluA1 prior to cLTP treatment, it completely blocked the activity-triggered increase we observed under control conditions, in agreement with previous studies demonstrating activity-triggered SEP-GluA1 trafficking from internal pools to the PM (Figure 2B) (Yudowski et al., 2007; Makino and Malinow, 2009; Petrini et al., 2009; Kennedy et al., 2010; Patterson et al., 2010). Despite the fact that total SEP-GluA1 signal slightly decreased following stimulation in TeNT-expressing neurons, we still observed rapid activity-triggered accumulation of SEP-GluA1 signal in dendritic spines (Figures 2C,D). Surprisingly, SEP-GluA1 accumulation 10-15 min following cLTP induction was indistinguishable from controls, ruling out a major role for regulated membrane trafficking during the initial phase of AMPA receptor recruitment to synaptic sites (Figure 2E). Initial activity-triggered spine growth was also unperturbed by TeNT (Figure 2D), consistent with previous work (Yang et al., 2008a). Importantly, increased spine SEP-GluA1 signal was not maintained in a significant fraction of spines from TeNT-expressing neurons, returning to baseline, pre-stimulation levels ~30 min following cLTP induction (Figures 2C–G, Video 2). Together these observations are consistent with membrane trafficking playing an essential role in maintaining receptors initially recruited to synaptic sites by lateral diffusion (Penn et al., 2017). While it is possible that some portion of the sustained synaptic SEP-GluA1 signal in control conditions is due to activity-triggered insertion of expressed receptors, we think that the previously reported internalization defects of SEP-GluA1 (Kennedy et al., 2010; Hiester et al., 2017) further support our interpretation that the majority of retained GluA1 comes from a pre-existing pool of surface receptors. Thus, we propose that the primary role of postsynaptic vesicle fusion during LTP is not to deliver new AMPA receptors to synapses, but to traffic unidentified factors that maintain accumulated synaptic receptors and stabilize spine growth (Figure 3). While there is abundant evidence that REs also deliver AMPA receptors to the PM, this pool may be more important for replenishing the “reserve pool” of extrasynaptic surface receptors critical for LTP (Granger et al., 2013).

### Future directions and outstanding questions

Recent work has begun to more critically interrogate the role of postsynaptic membrane trafficking during LTP and suggests that the role of activity-triggered membrane fusion extends beyond regulating trafficking of AMPA receptors. We speculate that intracellular vesicles, REs in particular, house a cocktail of important synaptic cargoes that can be mobilized to the neuronal PM in response to synaptic activation. We propose that activity-triggered delivery of these cargoes is critical for stabilizing synaptic AMPA receptors and spine growth during LTP (Figure 3). In this light, we think there are several fundamental gaps in our understanding of how membrane trafficking contributes to synaptic plasticity.

#### What proteins mediate AMPA receptor stability during LTP?

AMPA receptors interact with an expanding list of proteins, many of which can impact receptor function and interactions with PSD proteins (Schwenk et al., 2012, 2014; Garcia-Nafria et al., 2016). Several families of TARPs, are required for the synaptic incorporation of AMPA receptors (Hashimoto et al., 1999; Chen et al., 2000; Tomita et al., 2003; Wang et al., 2008a; for a detailed review see Jackson and Nicoll, 2011). In addition, several families of transmembrane cell adhesion molecules, several of which are implicated in LTP, interact with AMPA receptors, including cadherins (Bozdagi et al., 2000; Nuriya and Huganir, 2006; Saglietti et al., 2007; Heisler et al., 2014; Brigidi et al., 2015), integrins (Chan et al., 2006; Huang et al., 2006; Cingolani et al., 2008; Pozo et al., 2012), LRRTMs (de Wit et al., 2009; Soler-llavina et al., 2013; Bhouri et al., 2018), neuroligins (Heine et al., 2008b; Mondin et al., 2011; Aoto et al., 2013), SynDIGs (Kalashnikova et al., 2010; Chenaux et al., 2016; Matt et al., 2018), and IgsF11 (Jang et al., 2016). Activity-triggered delivery of these proteins to the postsynaptic membrane could provide a mechanism to retain laterally diffusing AMPA receptors during LTP or to stabilize receptors already recruited to and trapped in the PSD. Regulated trafficking of these molecules could also play a role in organizing trans-synaptic “nanocolumns” where postsynaptic receptors are precisely positioned opposite presynaptic neurotransmitter release sites (Tang et al., 2016). However, whether any of these proteins localize to REs or are trafficked to the PM in response to activity remains largely unknown, but a recent study demonstrating a major role for REs in forward trafficking through the biosynthetic secretory pathway suggests that a diverse array of integral membrane proteins and secreted factors could at least initially traffic through REs (Bowen et al., 2017). Interestingly, endosomal-mediated surface trafficking of N-cadherin is critical for neural migration (Kawauchi et al., 2010; Jossin and Cooper, 2011; Ye et al., 2014; Hara et al., 2016), indicating that N-cadherin function may be broadly regulated at the level of surface trafficking. N-cadherin stabilization on the cell surface may be further aided by palmitoylation and RE-dependent synaptic recruitment of the scaffolding protein δ-catenin (Brigidi et al., 2014, 2015). Moreover, surface levels of β3-integrin increase during homeostatic synaptic strengthening (Cingolani et al., 2008), highlighting the possibility that trafficking of important AMPA receptor interacting proteins may be coupled to synaptic activity. However, this does not appear to be the case for the canonical TARP stargazin, which does not appear to internalize with AMPA receptors following agonist-dependent endocytosis (Tomita et al., 2004). Additionally, LRRTM 1 and 2 are required for both basal AMPA receptor transmission and LTP, suggesting these molecules may play a role in AMPAR receptor recruitment during synapse formation and plasticity (Bhouri et al., 2018). Whether LRRTMs are mobilized to the PM through regulated membrane trafficking mechanisms to modulate synaptic AMPA receptor stability during LTP remains unknown.

In addition to integral membrane proteins, peripheral membrane-associated proteins also associate with REs. For example, AKAP79/150, a key scaffold protein involved in coordinating postsynaptic kinase and phosphatase signaling, localizes to REs via palmitoylation and an N-terminal polybasic region and is delivered to dendritic spines following cLTP stimulation via a RE-dependent mechanism (Keith et al., 2012; Woolfrey et al., 2015). Thus activity-triggered RE fusion with the PM in dendrites and spines would be expected to alter the subcellular distribution of signaling complexes that could directly or indirectly modify AMPA receptor localization and/or function.

It is also possible that secreted signaling molecules may mediate AMPA receptor stability. An intriguing recent study identified a critical role for Wnt signaling during the early stages of LTP. This study demonstrated that a specific Wnt protein, Wnt7a/b, rapidly accumulates at synapses in response to cLTP stimulation, and that Wnt7a/b promotes the diffusional trapping of AMPA receptors through activation of postsynaptic frizzled-7 (Mcleod et al., 2018). Whether Wnt7a/b secretion occurs from pre- and/or postsynaptic neurons remains to be determined. Brain derived neurotrophic factor (BDNF) has also been reported to be secreted from dendrites and directly from activated spines, where it could act in an autocrine manner through local activation of TrkB to support spine growth associated with LTP (Tanaka et al., 2008; Harward et al., 2016). Finally, proteases that remodel the extracellular matrix have been implicated in synaptic plasticity (Wang et al., 2008b; Szepesi et al., 2014). In particular, matrix metalloproteinase-9 (MMP-9) has been demonstrated to be both necessary and sufficient for morphological and functional LTP (Nagy et al., 2006; Bozdagi et al., 2007; Wang et al., 2008b).

Intriguingly, MMP-9-dependent plasticity itself requires postsynaptic exocytosis. Wang et al. (2008b) demonstrated that both synaptic and structural plasticity induced by application of exogenous MMP9 was blocked by loading neurons with botulinum toxin B (which cleaves VAMP proteins required for regulated secretion), suggesting that critical activators or important substrates of MMP-9 undergo postsynaptic exocytosis during LTP. Related, Padamsey et al. (2017) demonstrated that neural activity triggers fusion of lysosomes with the postsynaptic membrane, and activates MMP-9 via secretion of the protease cathepsin-B. It is unclear how neural activity is coupled to lysosomal membrane fusion, but Ca^2+^ release from the lysosomes themselves appears to play a critical role in promoting fusion, suggesting that there may be mechanisms for lysosomal fusion that are distinct from other vesicle types (Padamsey et al., 2017). An additional study demonstrated that lysosomes are distributed throughout neuronal dendrites and that activation of NMDA receptors recruits lysosomes to dendritic spines, further supporting a role for lysosome-mediated postsynaptic trafficking during synaptic plasticity (Goo et al., 2017). Whether lysosomes regulate trafficking of additional cargoes and whether lysosomal fusion with the PM utilizes shared mechanisms with other types of vesicles remains to be seen. Lysosomes could also be recruited near synaptic sites in response to activity to fulfill their more canonical role in protein turnover during times of increased synaptic remodeling.

Finally, it is also possible that AMPA receptors themselves act as stabilizing factors. AMPA receptors at hippocampal synapses are tetramers, primarily composed of GluA1/2 or GluA2/3 subunit assemblies (Lu et al., 2009; Traynelis et al., 2010). Previous work has demonstrated that immediately following LTP induction, GluA2-lacking AMPA receptors (which are high-conductance, inwardly rectifying and Ca^2+^ permeable) are incorporated into synapses (Plant et al., 2006). GluA2-lacking receptors are replaced by GluA2-containing receptors, with full exchange occurring ~25 min following LTP induction. This timescale is similar to the decay of excitatory postsynaptic potentials to baseline following LTP induction in the presence of various agents that block regulated membrane fusion reported in Lledo et al. (1998). Thus, it is possible that membrane fusion could deliver GluA2-containing receptors to synapses (or an unknown factor that promotes the exchange of GluA2-lacking for GluA2-containing AMPA receptors) in the minutes following LTP induction.

While the field has narrowly focused on AMPA receptors, identifying the full repertoire of synaptic proteins trafficked to the surface during LTP will be important for a comprehensive, mechanistic understanding of why postsynaptic membrane trafficking is essential for plasticity. One potential approach will be to perform quantitative proteomic measurements to identify those proteins whose surface localization is altered following global LTP stimuli and/or by blocking intracellular vesicle fusion with the PM. This approach was used to identify endosomal cargoes that contribute to cancer invasiveness and could potentially be applied to diverse forms of neuronal plasticity, although given the heterogeneity of neuronal and glial subtypes, whose surface proteome may behave differently, this approach may be challenging (Diaz-Vera et al., 2017). An alternative approach would be to specifically label proteins within organelles relevant for LTP to identify factors that are likely to traffic to the cell surface, or be secreted following synaptic stimulation (Hung et al., 2014). An advantage to this strategy is that it may be able to distinguish populations of proteins that are trafficked through different postsynaptic organelles (e.g., REs vs. lysosomes). In any case, defining the full spectrum of postsynaptic vesicular protein cargoes will be invaluable in understanding the relationship between membrane trafficking and spine growth/AMPA receptor stability during LTP.

#### How is synaptic activity coupled to vesicle fusion?

Strong synaptic activation associated with LTP drives robust fusion of intracellular vesicles with the postsynaptic membrane, but the precise mechanisms underlying how activity is coupled to membrane fusion are only now emerging. Several studies have identified many of the fusion proteins that are required for expression of diverse forms of plasticity (Gerges et al., 2006; Lin et al., 2009; Araki et al., 2010; Kennedy et al., 2010; Ahmad et al., 2012; Jurado et al., 2013; Arendt et al., 2015; Wu et al., 2017a; Bin et al., 2018). Additionally, work from our lab demonstrated that L-type voltage-gated Ca^2+^ channels (L-VGCCs) play an important modulatory role during RE fusion by regulating whether REs partially or fully fuse with the PM, thus providing a potential mechanism for regulating the factors released to the PM or extracellular space during RE fusion events (Hiester et al., 2017) (Figure 3). Research aimed at identifying additional proteins that regulate postsynaptic fusion could help uncover similarly complex regulatory mechanisms. Further, it is possible that this avenue of research may also demonstrate a requirement for postsynaptic membrane fusion in other types of plasticity. Indeed, a recent study by Arendt et al. (2015) identified a requirement for SNARE-mediated membrane fusion during retinoic acid-induced homeostatic plasticity, suggesting that regulation of AMPA receptor stability through postsynaptic exocytosis may be broadly important for diverse forms of plasticity.

#### What is the role of spine RE fusion?

A lingering controversy regards the extent to which vesicle fusion occurs in dendritic spines. Multiple studies have demonstrated that endosomes can fuse with the dendritic spine plasma membrane (Kennedy et al., 2010; Patterson et al., 2010; Hiester et al., 2017). What remains unknown and controversial is the extent to which these fusion events could deliver AMPA receptors or other factors that could contribute to synaptic potentiation during LTP. Blocking regulated membrane trafficking with TeNT does not impede initial spine SEP-GluA1 accumulation or spine growth, but leads to loss of accumulated receptors and reduction in spine size several minutes following induction (Figures 2C–G). While this experiment does not specifically test the role of spine RE fusion (since TeNT blocks fusion in spines and the shaft), delivery of protein cargoes directly within dendritic spines is likely to have a much different functional outcome than in dendritic shafts, so it will be important to resolve this issue. Current strategies for directly visualizing AMPA receptor trafficking in live cells, (e.g., SEP-GluA1) likely underestimate the full extent of spine AMPA receptor insertion, underscoring the need for tools that will allow visualization of endogenous AMPA receptor trafficking (Kennedy et al., 2010; Hiester et al., 2017). One method that has been used in several studies relies upon differential antibody labeling to selectively visualize AMPARs that are inserted into the membrane after stimulation (Lu et al., 2001; Sun et al., 2005; Hiester et al., 2017; Werner et al., 2017). However, a major limitation of this technique is that it lacks the requisite temporal specificity to precisely identify when and where receptors are inserted into the membrane. An alternative approach would be to chemically label endogenous AMPA receptors in live cells (Wakayama et al., 2017), though it remains to be seen whether such a technique can be adapted to specifically monitor discrete receptor trafficking events. Genetically encoded affinity tags against endogenous excitatory and inhibitory synaptic scaffold and signaling proteins have been extremely valuable tools for labeling synaptic structures (Gross et al., 2013, 2016; Mora et al., 2013; Barcomb et al., 2015; Fossati et al., 2016; Kannan et al., 2016; Son et al., 2016; Spence et al., 2016; Uezu et al., 2016; Goodell et al., 2017; Lin et al., 2017; Sinnen et al., 2017; Walker et al., 2017). Similar reagents for labeling endogenous AMPA receptors would be valuable for addressing numerous basic questions concerning how endogenous receptors traffic. As with any tagging strategy, targeting intrabodies to benign epitopes within AMPA receptors will be critical. Likewise, new tools that would allow spatially-restricted inhibition of specific organelle function (e.g., REs and lysosomes) within different subcellular domains will be invaluable for unraveling precisely where, when and how membrane fusion relevant for plasticity occurs (Bourke et al., 2018).

## Concluding remarks

The neuronal postsynaptic membrane is a dynamic structure that undergoes major changes during synaptic plasticity. During LTP, activity-triggered recruitment of AMPA receptors is one of the most critical alterations at the synaptic membrane for enduring plasticity. While the field has made great progress in understanding many of the underlying mechanisms of AMPA receptor trafficking, our understanding of how AMPA receptor surface delivery contributes to plasticity is considerably less clear. Recent work challenges the assumption that activity-triggered delivery of AMPA receptors to the PM plays a direct role in LTP. However, this work highlights the importance of postsynaptic membrane fusion beyond merely delivering AMPA receptors, forcing the field to generate new models for how membrane trafficking contributes to synaptic plasticity. We propose that postsynaptic membrane fusion delivers diverse proteins to the dendritic PM, some of which may be critical for stabilizing synaptic AMPA receptors during LTP, thus reconciling seemingly contradictory results in the field. Identifying the complete cast of proteins delivered to the cell surface during plasticity and the intracellular organelles responsible will help to reshape our understanding of how membrane trafficking impacts synaptic function and plasticity.

## Methods

### Cell culture and transfection

All animal procedures were carried out in accordance with a protocol approved by the Institutional Animal Care and Use Committee at the University of Colorado School of Medicine. Dissociated hippocampal cultures were prepared from neonatal rat pups as previously described (Beaudoin et al., 2012) and grown on 18 mm poly-D-lysine (Sigma) coated coverslips in 12-well cell culture dishes in Neurobasal-A medium (Invitrogen) supplemented with B27 (Invitrogen) and Glutamax (Invitrogen) at an approximate density of 100,000 cells/well. Neurons were maintained at 37°C in a humidified incubator at 5% CO_2_. All neurons were between DIV18 and DIV21 at the time of experiment.

Neurons were transfected using Lipofectamine 2000 (Invitrogen) according to the manufacturer's recommendations and allowed to express plasmids for 48–72 h prior to experiments. For all experiments, neurons were transfected with a plasmid encoding the AMPA receptor subunit GluA1 tagged N-terminally with superecliptic pHluorin (SEP) (Kennedy et al., 2010). For control conditions, neurons were transfected with a plasmid encoding soluble mCh. For the tetanus toxin (TeNT) condition, neurons were transfected with a bicistronic plasmid encoding mCh fused to TeNT with a cleavable P2A peptide tag (mCh-P2A-TeNT) (Szymczak et al., 2004).

### Image acquisition and data analysis

Live cell imaging of dissociated neurons was carried out at 32°C on an Olympus IX71 equipped with a spinning disc scan head (Yokogawa). Excitation illumination was delivered from an acousto-optic tunable filter (AOTF) controlled laser launch (Andor). Images were acquired using a 60x Plan Apochromat 1.4 NA objective and collected on a 1024 × 1024 pixel Andor iXon EM-CCD camera. For all imaging experiments, the apical portion of the dendrtic arbor extending 25–100 μm from the cell soma was imaged. Data acquisition and analysis were performed with Metamorph (Molecular Devices) and ImageJ software. Some images were low pass filtered and interpolated for display. Only raw, unprocessed data were used for quantification.

To image activity-triggered SEP-GluA1 exocytosis and SEP-GluA1 translocation, transfected neurons were pretreated with tetrodotoxin (TTX, Tocris, 1–2 μM) for 1 h to inhibit evoked activity. Coverslips with cultured neurons were then placed in a live-cell imaging chamber (Ludin) and incubated in baseline ACSF solution containing (in mM): 130 NaCl, 5 KCl, 10 HEPES, 30 glucose, 1 MgCl_2_, 2 CaCl_2_, and 0.002 TTX (pH 7.4). To stimulate synaptic activity (cLTP stimulation), the baseline solution was exchanged for one that contained (in mM): 130 NaCl, 5 KCl, 10 HEPES, 30 glucose, 0 MgCl_2_, 2 CaCl_2_, 0 TTX, and 0.2 glycine. After cLTP stimulation, neurons were re-exposed to the baseline solution for the remainder of the imaging period.

To measure discrete SEP-GluA1 exocytosis events, single plane 2-color (SEP-GluA1, TfRmCh/dsred-homer1c/PSD95-mCh) images were acquired at 2 Hz. For measuring SEP-GluA1 surface delivery and spine morphology, 2-color (mCh, SEP-GluA1) 5 μM z-stacks were acquired every 1 min before, during and after bath stimulation. To quantify the rate of synaptic SEP-GluA1 accumulation circular ROIs were drawn over individual dendritic spine heads and the mean background-subtracted SEP-GluA1 signal was quantified in ImageJ. To measure the peak synaptic SEP-GluA1 accumulation in an unbiased manner (Figure 1C), ROIs were drawn over randomly selected dendritic spine heads using the mCh signal without regard to the amount of cLTP-induced SEP-GluA1 accumulation, and the average SEP-GluA1 signal between 10 and 15 mins post cLTP was calculated for each spine. To selectively measure the retainment of SEP-GluA1 after cLTP (Figures 1D,E), ROIs were drawn over dendritic spine heads that displayed at least a 25% increase in SEP-GluA1 accumulation over baseline. Data are plotted as the ratio of SEP-GluA1 fluorescence at any given time point over the SEP-GluA1 fluorescence at the start of the experiment (SEP-GluA1 F/F_0_).

Statistical significance for experiments comparing the means of two populations was determined using a two-tailed unpaired Student's *t*-test. In cases where measurements of two populations were recorded over multiple time points, a two-way ANOVA with Bonferroni multiple comparison test was used or a two-sample Kolmogorov-Smirnov test.

## Author contributions

BH, MB, AB, and MK contributed to data collection, analysis and interpretation. SS contributed to data analysis and interpretation. BH, and MK wrote and edited the manuscript.

### Conflict of interest statement

The authors declare that the research was conducted in the absence of any commercial or financial relationships that could be construed as a potential conflict of interest.

## Supplementary material

Supplementary Figure 1 ER acidification suppresses SEP-GluA1 signal during cLTP. **(A)** Method for masking the dendritic shaft for SEP-GluA1 signal analysis. A mask was generated so that the dendritic shaft signal could be selectively quantified. This was generated by subtracting a synapse mask, based on the SEP-GluA1 signal from a whole cell mask generated from the mCh cell fill (see methods below for details). Shown is the mCh cell fill (panel i), binary synapse mask based on the SEP-GluA1 signal (red, panel ii) and the SEP-GluA1 signal (gray scale, panel iii) with dendritic shaft mask overlayed (burgundy). Scale bar 10 μm. **(B)** Quantification of the spine (solid line) and shaft (dashed line) SEP-GluA1 signal as a function of time during and after cLTP (gray bar). Note the robust decrease in shaft signal, which slightly precedes synaptic accumulation. Error bars represent standard error of the mean.This decrease is likely due to ER acidification as outlined in Rathje et al. (2013).

Video 1 SEP-GluA1 stably accumulates at dendritic spines in response to cLTP. Representative dendritic segment from a control neuron showing SEP-GluA1 (**Top**), mCh (**Middle**), and a merge of both channels (**Bottom**). The white dot in the bottom right indicates the duration of the cLTP stimulus. Asterisks mark dendritic spines displaying stable accumulation of SEP-GluA1 and spine growth. The duration of the video is 55 min with an acquisition rate of 1 image/min, and played back at 10 frames *per sec*.

Video 2 SEP-GluA1 transiently accumulates at a subset of dendritic spines following cLTP when postsynaptic membrane fusion is blocked. Representative dendritic segment from a TeNT expressing neuron showing SEP-GluA1 (**Top**), mCh (**Middle**), and a merge of both channels (**Bottom**). The white dot in the bottom right indicates the duration of the cLTP stimulus. Asterisks mark dendritic spines displaying initial accumulation of SEP-GluA1 and spine growth that is not stably maintained throughout the experiment. The duration of the video is 55 min with an acquisition rate of 1 image/min, and played back at 10 frames *per sec*.

## References

[B1] Ahmad M. Polepalli J. S. Goswami D. Yang X. Kaeser-Woo Y. J. Südhof T. C.. (2012). Postsynaptic complexin controls AMPA receptor exocytosis during LTP. Neuron 73, 260–267. 10.1016/j.neuron.2011.11.020 22284181 PMC3269030

[B2] Aoto J. Martinelli D. C. Malenka R. C. Tabuchi K. Su T. C. (2013). Presynaptic neurexin-3 alternative splicing trans-synaptically controls postsynaptic AMPA receptor trafficking. Cell 154, 75–88. 10.1016/j.cell.2013.05.060 23827676 PMC3756801

[B3] Araki Y. Lin D. Huganir R. L. (2010). Plasma membrane insertion of the AMPA receptor GluA2 subunit is regulated by NSF binding and Q/R editing of the ion pore. Proc. Natl. Acad. Sci. U.S.A. 107, 11080–11085. 10.1073/pnas.1006584107 20534470 PMC2890737

[B4] Arendt K. L. Zhang Y. Jurado S. Malenka R. C. Südhof T. C. Chen L. (2015). Retinoic acid and LTP recruit postsynaptic AMPA receptors using distinct SNARE-dependent mechanisms. Neuron 86, 442–456. 10.1016/j.neuron.2015.03.009 25843403 PMC4578641

[B5] Ashby M. C. Maier S. R. Nishimune A. Henley J. M. (2006). Lateral diffusion drives constitutive exchange of AMPA receptors at dendritic spines and is regulated by spine morphology. J. Neurosci. 26, 7046–7055. 10.1523/JNEUROSCI.1235-06.2006 16807334 PMC6673929

[B6] Banke T. G. Bowie D. Lee H. Huganir R. L. Schousboe A. Traynelis S. F. (2000). Control of GluR1 AMPA receptor function by cAMP-dependent protein kinase. J. Neurosci. 20, 89–102. 10.1523/JNEUROSCI.20-01-00089.2000 10627585 PMC6774102

[B7] Barcomb K. Goodell D. J. Arnold D. B. Bayer U. (2015). Live imaging of endogenous Ca^2+^/calmodulin-dependent protein kinase II in neurons reveals that ischemia-related aggregation does not require kinase activity. J. Neurochem. 135, 666–673. 10.1111/jnc.13263 26212614 PMC4636925

[B8] Barria A. Muller D. Derkach V. Griffith L. C. Soderling T. R. (1997). Regulatory phosphorylation of AMPA-type glutamate receptors by CaM-KII during long-term potentiation. Science 276, 2042–2046. 10.1126/science.276.5321.2042 9197267

[B9] Bats C. Groc L. Choquet D. (2007). Article the interaction between stargazin and PSD-95 regulates AMPA receptor surface trafficking. Neuron 53, 719–734. 10.1016/j.neuron.2007.01.030 17329211

[B10] Beaudoin G. M. J. Lee S. H. Singh D. Yuan Y. Ng Y. G. Reichardt L. F.. (2012). Culturing pyramidal neurons from the early postnatal mouse hippocampus and cortex. Nat. Protoc. 7, 1741–1754. 10.1038/nprot.2012.099 22936216

[B11] Benke T. Traynelis S. F. (2018). AMPA-type glutamate receptor conductance changes and plasticity: still a lot of noise. Neurochem. Res. [Epub ahead of print]. 10.1007/s11064-018-2491-1 29476449 PMC6533624

[B12] Benke T. A. Luthi A. Isaac J. T. R. Collingridge G. L. (1998). Modulation of AMPA receptor unitary conductance by synaptic activity. Nature 393, 793–797. 10.1038/31709 9655394

[B13] Bhouri M. Morishita W. Temkin P. Goswami D. Kawabe H. Brose N.. (2018). Deletion of LRRTM1 and LRRTM2 in adult mice impairs basal AMPA receptor transmission and LTP in hippocampal CA1 pyramidal neurons. Proc. Natl. Acad. Sci. U.S.A. 115, E5382–E5389. 10.1073/pnas.1803280115 29784826 PMC6003336

[B14] Biederer T. Kaeser P. S. Blanpied T. A. (2017). Review transcellular nanoalignment of synaptic function. Neuron 96, 680–696. 10.1016/j.neuron.2017.10.006 29096080 PMC5777221

[B15] Bin N. Ma K. Harada H. Tien C.-W. Bergin F. Sugita K. (2018). Crucial role of postsynaptic syntaxin 4 in mediating basal neurotransmission and synaptic plasticity in article crucial role of postsynaptic syntaxin 4 in mediating basal neurotransmission and synaptic plasticity in hippocampal CA1 neurons. Cell Rep. 23, 2955–2966. 10.1016/j.celrep.2018.05.026 29874582

[B16] Bliss T. V. P. Collingridge G. L. (1993). A synaptic model of memory: long-term potentiation in the hippocampus. Nature 361, 31–39. 10.1038/361031a0 8421494

[B17] Borgdorff A. J. Choquet D. (2002). Regulation of AMPA receptor lateralmovements. Nature 369, 649–653. 10.1038/nature00780 12050666

[B18] Bourke A. M. Bowen A. B. Kennedy M. J. (2018). New approaches for solving old problems in neuronal protein trafficking. Mol. Cell. Neurosci. 91, 48–66. 10.1016/j.mcn.2018.04.004 29649542 PMC6128763

[B19] Bowen A. B. Bourke A. M. Hiester B. G. Hanus C. Kennedy M. J. (2017). Golgi-independent secretory trafficking through recycling endosomes in neuronal dendrites and spines. Elife 6:e27362. 10.7554/eLife.27362 28875935 PMC5624785

[B20] Bozdagi O. Nagy V. Kwei K. T. Huntley G. W. (2007). *In vivo* roles for matrix metalloproteinase-9 in mature hippocampal synaptic physiology and plasticity. J. Neurophysiol. 98, 334–344. 10.1152/jn.00202.2007 17493927 PMC4415272

[B21] Bozdagi O. Shan W. Tanaka H. Benson D. L. Huntley G. W. (2000). Increasing numbers of synaptic puncta during late- phase LTP: N-cadherin is synthesized, recruited to synaptic sites, and required for potentiation. Neuron 28, 245–259. 10.1016/S0896-6273(00)00100-8 11086998

[B22] Brigidi G. S. Santyr B. Shimell J. Jovellar B. Bamji S. X. (2015). Activity-regulated trafficking of the palmitoyl-acyl transferase DHHC5. Nat. Commun. 6:8200. 10.1038/ncomms9200 26334723 PMC4569850

[B23] Brigidi G. S. Sun Y. Beccano-Kelly D. Pitman K. Mobasser M. Borgland S. L.. (2014). Palmitoylation of δ-catenin by DHHC5 mediates activity-induced synapse plasticity. Nat. Neurosci. 17, 522–532. 10.1038/nn.3657 24562000 PMC5025286

[B24] Broutman G. Baudry M. (2001). Involvement of the secretory pathway for AMPA receptors in NMDA-induced potentiation in hippocampus. J. Neurosci. 21, 27–34. 10.1523/JNEUROSCI.21-01-00027.2001 11150316 PMC6762437

[B25] Brown T. C. Correia S. S. Petrok C. N. Esteban J. A. (2007). Functional compartmentalization of endosomal trafficking for the synaptic delivery of AMPA receptors during long-term potentiation. J. Neurosci. 27, 13311–13315. 10.1523/JNEUROSCI.4258-07.2007 18045925 PMC6673392

[B26] Chan C.-S. Weeber E. J. Zong L. Fuchs E. Sweatt J. D. Davis R. L. (2006). Beta1-integrins are required for hippocampal AMPA receptor-dependent synaptic transmission, synaptic plasticity, and working memory. J. Neurosci. 26, 223–232. 10.1523/JNEUROSCI.4110-05.2006 16399691 PMC2408376

[B27] Chen L. Chetkovich D. M. Petralia R. S. Sweeney N. T. Kawasaki Y. Wenthold R. J.. (2000). Stargazin regulates synaptic targeting of AMPA receptors by two distinct mechanisms. Nature 408, 936–943. 10.1038/35050030 11140673

[B28] Chenaux G. Matt L. Hill T. C. Kaur I. Liu X. Kirk L. M. (2016). Loss of SynDIG1 reduces excitatory synapse maturation but not formation *in vivo*. ENeuro 3, 1–17. 10.1523/ENEURO.0130-16.2016 PMC5073248 27800545

[B29] Cingolani L. A. Thalhammer A. Yu L. M. Y. Catalano M. Ramos T. Colicos M. A.. (2008). Activity-dependent regulation of synaptic AMPA receptor composition and abundance by β3 integrins. Neuron 58, 749–762. 10.1016/j.neuron.2008.04.011 18549786 PMC2446609

[B30] Cooney J. R. Hurlburt J. L. Selig D. K. Harris K. M. Fiala J. C. (2002). Endosomal compartments serve multiple hippocampal dendritic spines from a widespread rather than a local store of recycling membrane. J. Neurosci. 22, 2215–2224. 10.1523/JNEUROSCI.22-06-02215.2002 11896161 PMC6758269

[B31] Derkach V. Barria A. Soderling T. R. (1999). Ca^2+^/calmodulin-kinase II enhances channel conductance of alpha-amino-3-hydroxy-5-methyl-4-isoxazolepropionate type glutamate receptors. Proc. Natl. Acad. Sci. U.S.A. 96, 3269–3274. 10.1073/pnas.96.6.3269 10077673 PMC15931

[B32] de Wit J. Sylwestrak E. O'Sullivan M. L. Otto S. Tiglio K. Savas J. N.. (2009). LRRTM2 interacts with neurexin1 and regulates excitatory synapse formation. Neuron 64, 799–806. 10.1016/j.neuron.2009.12.019 20064388 PMC2829666

[B33] Díaz-Alonso J. Sun Y. J. Granger A. J. Levy J. M. Blankenship S. M. Nicoll R. A. (2017). Subunit-specific role for the amino-terminal domain of AMPA receptors in synaptic targeting. Proc. Natl. Acad. Sci.U.S.A. 114, 7136–7141. 10.1073/pnas.1707472114 28630296 PMC5502653

[B34] Diaz-Vera J. Palmer S. Hernandez-Fernaud J. R. Dornier E. Mitchell L. E. Macpherson I.. (2017). A proteomic approach to identify endosomal cargoes controlling cancer invasiveness. J. Cell Sci. 130, 697–711. 10.1242/jcs.190835 28062852 PMC5339883

[B35] Ehlers M. D. (2000). Reinsertion or degradation of AMPA receptors determined by activity-dependent endocytic sorting. Neuron 28, 511–525. 10.1016/S0896-6273(00)00129-X 11144360

[B36] Ehlers M. D. Heine M. Groc L. Lee M. Choquet D. (2007). Diffusional trapping of GluR1 AMPA receptors by input-specific synaptic activity. Neuron 54, 447–460. 10.1016/j.neuron.2007.04.010 17481397 PMC1993808

[B37] Ehrlich I. Klein M. Rumpel S. Malinow R. (2007). PSD-95 is required for activity-driven synapse stabilization. Proc. Natl. Acad. Sci. U.S.A. 104, 4176–4181. 10.1073/pnas.0609307104 17360496 PMC1820728

[B38] Esteves da Silva M. Adrian M. Schätzle P. Lipka J. Watanabe T. Cho S.. (2015). Positioning of AMPA receptor-containing endosomes regulates synapse architecture. Cell Rep. 13, 933–943. 10.1016/j.celrep.2015.09.062 26565907

[B39] Fossati M. Pizzarelli R. Schmidt E. R. Kupferman J. V. Stroebel D. Polleux F.. (2016). Regulate the development of excitatory and inhibitory synapses SRGAP2 and its human-specific paralog co-regulate the development of excitatory and inhibitory synapses. Neuron 91, 356–369. 10.1016/j.neuron.2016.06.013 27373832 PMC5385270

[B40] Garcia-Nafria J. Herguedas B. Watson J. F. Greger I. H. (2016). The dynamic AMPA receptor extracellular region: a platform for synaptic protein interactions. J. Physiol. 19, 5449–5458. 10.1113/JP271844 PMC5043031 26891027

[B41] Gerges N. Z. Backos D. S. Rupasinghe C. N. Spaller M. R. Esteban J. A. (2006). Dual role of the exocyst in AMPA receptor targeting and insertion into the postsynaptic membrane. EMBO J. 25, 1623–1634. 10.1038/sj.emboj.7601065 16601687 PMC1440842

[B42] Goo M. S. Sancho L. Slepak N. Boassa D. Deerinck T. J. Ellisman M. H.. (2017). Activity-dependent trafficking of lysosomes in dendrites and dendritic spines. J. Neurosci. 216, 2499–2513. 10.1083/jcb.201704068 28630145 PMC5551717

[B43] Goodell D. J. Zaegel V. Coultrap S. J. Hell J. W. Bayer K. U. Goodell D. J.. (2017). DAPK1 mediates LTD by making CaMKII/GluN2B binding LTP specific. Cell Rep. 19, 2231–2243. 10.1016/j.celrep.2017.05.068 28614711 PMC5549467

[B44] Granger A. J. Shi Y. Lu W. Cerpas M. Nicoll R. A. (2013). LTP requires a reserve pool of glutamate receptors independent of subunit type. Nature 493, 495–500. 10.1038/nature11775 23235828 PMC3998843

[B45] Groc L. Heine M. Cognet L. Brickley K. Stephenson F. A. Lounis B.. (2004). Differential activity-dependent regulation of the lateral mobilities of AMPA and NMDA receptors. Nat. Neurosci. 7, 695–696. 10.1038/nn1270 15208630

[B46] Gross G. G. Junge J. A. Mora R. J. Kwon H. Olson C. A. Takahashi T. T.. (2013). Recombinant probes for visualizing endogenous synaptic proteins in living neurons. Neuron 78, 971–985. 10.1016/j.neuron.2013.04.017 23791193 PMC3779638

[B47] Gross G. G. Straub C. Perez-sanchez J. Dempsey W. P. Junge J. A. Roberts R. W.. (2016). An E3-ligase-based method for ablating inhibitory synapses. Nat. Methods 13, 673–678. 10.1038/nmeth.3894 27271196 PMC5312699

[B48] Hanus C. Kochen L. Tom Dieck S. Racine V. Sibarita J. B. Schuman E. M.. (2014). Synaptic control of secretory trafficking in dendrites. Cell Rep. 7, 1771–1778. 10.1016/j.celrep.2014.05.028 24931613 PMC5321479

[B49] Hara Y. Fukaya M. Hayashi K. Kawauchi T. Nakajima K. Sakagami H. (2016). ADP ribosylation factor 6 regulates neuronal migration in the developing cerebral cortex through FIP3/arfophilin-1-dependent endosomal trafficking of N-cadherin. ENeuro 3:ENEURO.0148–16.2016. 10.1523/ENEURO.0148-16.2016 27622210 PMC5002984

[B50] Harward S. C. Hedrick N. G. Hall C. E. Parra-Bueno P. Milner T. A. Pan E.. (2016). Autocrine BDNF-TrkB signalling within a single dendritic spine. Nature 538, 99–103. 10.1038/nature19766 27680698 PMC5398094

[B51] Hashimoto K. Fukaya M. Qiao X. Sakimura K. Watanabe M. Kano M. (1999). Impairment of AMPA receptor function in cerebellar granule cells of ataxic mutant mouse stargazer. J. Neurosci. 19, 6027–6036. 10.1523/JNEUROSCI.19-14-06027.1999 10407040 PMC6783074

[B52] Hayashi Y. Shi S.-H. Esteban J. A. Piccini A. Poncer J.-C. Malinow R. (2000). Driving AMPA receptors into synapses by LTP and CaMKII: requirement for GluR1 and PDZ domain interaction. Science 287, 2262–2268. 10.1126/science.287.5461.2262 10731148

[B53] Heine M. Groc L. Frischknecht R. Beique J. C. Lounis B. Rumbaugh G.. (2008a). Surface mobility of postsynaptic AMPARs tunes synaptic transmission. Science 320, 201–205. 10.1126/science.1152089 18403705 PMC2715948

[B54] Heine M. Thoumine O. Mondin M. Tessier B. Giannone G. Choquet D. (2008b). Activity-independent and subunit-specific recruitment of functional AMPA receptors at neurexin/neuroligin contacts. Proc. Natl. Acad. Sci. U.S.A. 105, 20947–20952. 10.1073/pnas.0804007106 19098102 PMC2634880

[B55] Heisler F. F. Lee H. K. Gromova K. V. Pechmann Y. Schurek B. Ruschkies L.. (2014). GRIP1 interlinks N-cadherin and AMPA receptors at vesicles to promote combined cargo transport into dendrites. Proc. Natl. Acad. Sci. U.S.A. 111, 5030–5035. 10.1073/pnas.1304301111 24639525 PMC3977296

[B56] Heynen A. J. Quinlan E. M. Bae D. C. Bear M. F. (2000). Bidirectional, activity-dependent regulation of glutamate receptors in the adult hippocampus *in vivo*. Neuron 28, 527–536. 10.1016/S0896-6273(00)00130-6 11144361

[B57] Hiester B. G. Bourke A. M. Sinnen B. L. Cook S. G. Gibson E. S. Smith K. R.. (2017). L-Type voltage-gated Ca^2+^channels regulate synaptic-activity-triggered recycling endosome fusion in neuronal dendrites. Cell Rep. 21, 2134–2146. 10.1016/j.celrep.2017.10.105 29166605 PMC6466620

[B58] Hruska M. Henderson N. Marchand S. J. Le J. H. Dalva M. B. (2018). Synaptic nanomodules underlie the organization and plasticity of spine synapses. Nat. Neurosci. 21, 671–682. 10.1038/s41593-018-0138-9 29686261 PMC5920789

[B59] Huang Z. Shimazu K. Woo N. H. Zang K. Mu U. Lu B.. (2006). Distinct roles of the beta-1-class integrins at the developing and the mature hippocampal excitatory synapse. J. Neurosci. 26, 11208–11219. 10.1523/JNEUROSCI.3526-06.2006 17065460 PMC2693048

[B60] Hung V. Zou P. Rhee H.-W. Udeshi N. D. Cracan V. Svinkina T.. (2014). Proteomic mapping of the human mitochondrial intermembrane space in live cells via ratiometric APEX tagging. Mol. Cell 55, 332–341. 10.1016/j.molcel.2014.06.003 25002142 PMC4743503

[B61] Isaac J. T. R. Nicoll R. A. Malenka R. C. (1995). Evidence for silent synapses: implications for the expression of LTP. Neuron 15, 427–434. 10.1016/0896-6273(95)90046-2 7646894

[B62] Jaafari N. Henley J. M. Hanley J. G. (2012). PICK1 mediates transient synaptic expression of GluA2- lacking AMPA receptors during glycine-induced AMPA receptor trafficking. J. Neurosci. 32, 11618–11630. 10.1523/JNEUROSCI.5068-11.2012 22915106 PMC6703756

[B63] Jaafari N. Konopacki F. A. Owen T. F. Kantamneni S. Rubin P. Craig T. J.. (2013). SUMOylation is required for glycine-induced increases in AMPA receptor surface expression (ChemLTP) in hippocampal neurons. PLoS ONE 8:e52345. 10.1371/journal.pone.0052345 23326329 PMC3543417

[B64] Jackson A. C. Nicoll R. A. (2011). The expanding social network of ionotropic glutamate receptors: TARPs and other transmembrane auxiliary subunits. Neuron 70, 178–199. 10.1016/j.neuron.2011.04.007 21521608 PMC3119519

[B65] Jang S. Oh D. Lee Y. Hosy E. Shin H. Van Riesen C.. (2016). Synaptic adhesion molecule IgSF11 regulates synaptic transmission and plasticity. Nat. Neurosci. 19, 84–93. 10.1038/nn.4176 26595655 PMC5010778

[B66] Jossin Y. Cooper J. A. (2011). Reelin, Rap1 and N-cadherin orient the migration of multipolar neurons in the developing neocortex. Nat. Neurosci. 14, 697–704. 10.1038/nn.2816 21516100 PMC3102785

[B67] Jurado S. Goswami D. Zhang Y. Molina A. J. M. Südhof T. C. Malenka R. C. (2013). LTP requires a unique postsynaptic SNARE fusion machinery. Neuron 77, 542–558. 10.1016/j.neuron.2012.11.029 23395379 PMC3569727

[B68] Kalashnikova E. Lorca R. A. Kaur I. Barisone G. A. Li B. Ishimaru T.. (2010). SynDIG1: an activity-regulated, AMPA- receptor-interacting transmembrane protein that regulates excitatory synapse development. Neuron 65, 80–93. 10.1016/j.neuron.2009.12.021 20152115 PMC2822728

[B69] Kannan M. Gross G. G. Arnold X. D. B. Higley M. J. (2016). Visual deprivation during the critical period enhances layer 2/3 GABAergic inhibition in mouse V1. J. Neurosci. 36, 5914–5919. 10.1523/JNEUROSCI.0051-16.2016 27251614 PMC4887562

[B70] Kawauchi T. Sekine K. Shikanai M. Chihama K. Tomita K. Kubo K. (2010). Article Rab GTPases-dependent endocytic pathways regulate neuronal migration and maturation through N-cadherin trafficking. Neuron 67, 588–602. 10.1016/j.neuron.2010.07.007 20797536

[B71] Keith D. J. Sanderson J. L. Gibson E. S. Woolfrey K. M. Robertson H. R. Olszewski K.. (2012). Palmitoylation of A-kinase anchoring protein 79/150 regulates dendritic endosomal targeting and synaptic plasticity mechanisms. J. Neurosci. 32, 7119–7136. 10.1523/JNEUROSCI.0784-12.2012 22623657 PMC3367663

[B72] Kennedy M. J. Davison I. G. Robinson C. G. Ehlers M. D. (2010). Syntaxin-4 defines a domain for activity-dependent exocytosis in dendritic spines. Cell 141, 524–535. 10.1016/j.cell.2010.02.042 20434989 PMC2874581

[B73] Kopec C. D. Li B. Wei W. Boehm J. Malinow R. (2006). Glutamate receptor exocytosis and spine enlargement during chemically induced long-term potentiation. J. Neurosci. 26, 2000–2009. 10.1523/JNEUROSCI.3918-05.2006 16481433 PMC6674938

[B74] Kopec C. D. Real E. Kessels H. W. Malinow R. (2007). GluR1 links structural and functional plasticity at excitatory synapses. J. Neurosci. 27, 13706–13718. 10.1523/JNEUROSCI.3503-07.2007 18077682 PMC6673607

[B75] Lang C. Barco A. Zablow L. Kandel E. R. Siegelbaum S. A. Zakharenko S. S. (2004). Transient expansion of synaptically connected dendritic spines upon induction of hippocampal long-term potentiation. Proc. Natl. Acad. Sci. U.S.A. 101, 16665–16670. 10.1073/pnas.0407581101 15542587 PMC534531

[B76] Liao D. Hessler N. Malinow R. (1995). Activation of postsynaptically silent synapses during pairing-induced LTP in CA1 region of hippocampal slice. Nature 375, 400–404. 10.1038/375400a0 7760933

[B77] Lin D. Makino Y. Sharma K. Hayashi T. Neve R. Takamiya K.. (2009). Regulation of AMPA receptor extrasynaptic insertion by 4.1N, phosphorylation and palmitoylation. Nat. Neurosci. 12, 879–887. 10.1038/nn.2351 19503082 PMC2712131

[B78] Lin L. Lo L. H-Y. Lyu Q. Lai K.-O. (2017). Determination of dendritic spine morphology by the striatin scaffold protein STRN4 through interaction with the phosphatase PP2A. J. Biol. Chem. 292, 9451–9464. 10.1074/jbc.M116.772442 28442576 PMC5465475

[B79] Lledo P. M. Zhang X. Südhof T. C. Malenka R. C. Nicoll R. A. (1998). Postsynaptic membrane fusion and long-term potentiation. Science 279, 399–403. 10.1126/science.279.5349.399 9430593

[B80] Lu W. Man H. Ju W. Trimble W. S. MacDonald J. F. Wang Y. T. (2001). Activation of synaptic NMDA receptors induces membrane insertion of new AMPA receptors and LTP in cultured hippocampal neurons. Neuron 29, 243–254. 10.1016/S0896-6273(01)00194-5 11182095

[B81] Lu W. Shi Y. Jackson A. C. Bjorgan K. During M. J. Sprengel R.. (2009). Subunit composition of synaptic AMPA receptors revealed by a single-cell genetic approach. Neuron 62, 254–268. 10.1016/j.neuron.2009.02.027 19409270 PMC3632349

[B82] MacGillavry H. D. Song Y. Raghavachari S. Blanpied T. A. (2013). Nanoscale scaffolding domains within the postsynaptic density concentrate synaptic AMPA receptors. Neuron 78, 615–622. 10.1016/j.neuron.2013.03.009 23719161 PMC3668352

[B83] Makino H. Malinow R. (2009). AMPA receptor incorporation into synapses during LTP: the role of lateral movement and exocytosis. Neuron 64, 381–390. 10.1016/j.neuron.2009.08.035 19914186 PMC2999463

[B84] Malenka R. C. Nicoll R. A. (1999). Long-term potentiation—a decade of progress? Science 285, 1870–1875. 10.1126/science.285.5435.1870 10489359

[B85] Maletic-Savatic M. Malinow R. (1998). Calcium-evoked dendritic exocytosis in cultured hippocampal neurons. Part I: Trans-Golgi Network-derived organelles undergo regulated exocytosis. J. Neurosci. 18, 6803–6813. 10.1523/JNEUROSCI.18-17-06803.1998 9712651 PMC6792980

[B86] Matsuzaki M. Honkura N. Ellis-Davies G. C. R. Kasai H. (2004). Structural basis of long-trm potentiation in single dendritic spines. Nature 429, 761–766. 10.1038/nature02617 15190253 PMC4158816

[B87] Matt L. Kirk L. M. Chenaux G. Crawley J. N. Hell J. W. D.i E. (2018). SynDIG4/Prrt1 is required for excitatory synapse development and plasticity underlying cognitive function. Cell Rep. 22, 2246–2253. 10.1016/j.celrep.2018.02.026 29490264 PMC5856126

[B88] Maxfield F. R. McGraw T. E. (2004). Endocytic recycling. Nat. Rev. Mol. Cell Biol. 5, 121–132. 10.1038/nrm1315 15040445

[B89] Mcleod F. Bossio A. Marzo A. Ciani L. Sibilla S. Hannan S.. (2018). Wnt signaling mediates LTP-dependent spine plasticity and AMPAR localization through frizzled-7 receptors. Cell Rep. 23, 1060–1071. 10.1016/j.celrep.2018.03.119 29694885 PMC5946458

[B90] Miesenbock G. De Angelis D. A. Rothman J. E. (1998). Visualizing secretion and synaptic transmission with pH-sensitive green fluorescent proteins. Nature 394, 192–195. 10.1038/28190 9671304

[B91] Mondin M. Labrousse V. Hosy E. Heine M. Levet F. Poujol C.. (2011). Neurexin-neuroligin adhesions capture surface-diffusing AMPA receptors through PSD-95 scaffolds. J. Neurosci. 31, 13500–13515. 10.1523/JNEUROSCI.6439-10.2011 21940442 PMC6623291

[B92] Mora R. J. Roberts R. W. Arnold D. B. (2013). Recombinant probes reveal dynamic localization of CaMKII_within somata of cortical neurons. J. Neurosci. 33, 14579–14590. 10.1523/JNEUROSCI.2108-13.2013 24005308 PMC3761057

[B93] Nagy V. Bozdagi O. Matynia A. Balcerzyk M. Okulski P. Dzwonek J.. (2006). Matrix metalloproteinase-9 is required for hippocampal late-phase long-term potentiation and memory. J. Neurosci. 26, 1923–1934. 10.1523/JNEUROSCI.4359-05.2006 16481424 PMC4428329

[B94] Nicoll R. A. (2017). A brief history of long-term potentiation. Neuron 93, 281–290. 10.1016/j.neuron.2016.12.015 28103477

[B95] Nuriya M. Huganir R. L. (2006). Regulation of AMPA receptor trafficking by N-cadherin. J. Neurochem. 97, 652–661. 10.1111/j.1471-4159.2006.03740.x 16515543

[B96] Opazo P. Labrecque S. Tigaret C. M. Frouin A. Wiseman P. W. De Koninck P.. (2010). CaMKII triggers the diffusional trapping of surface AMPARs through phosphorylation of stargazin. Neuron 67, 239–252. 10.1016/j.neuron.2010.06.007 20670832

[B97] Padamsey Z. McGuinness L. Bardo S. J. Reinhart M. Tong R. Hedegaard A.. (2017). Activity-dependent exocytosis of lysosomes regulates the structural plasticity of dendritic spines. Neuron 93, 132–146. 10.1016/j.neuron.2016.11.013 27989455 PMC5222721

[B98] Park M. Penick E. C. Edwards J. G. Kauer J. A. Ehlers M. D. (2004). Recycling endosomes supply AMPA receptors for LTP. Science 305, 1972–1975. 10.1126/science.1102026 15448273

[B99] Park M. Salgado J. M. Ostroff L. Helton T. D. Robinson C. G. Harris K. M.. (2006). Plasticity-induced growth of dendritic spines by exocytic trafficking from recycling endosomes. Neuron 52, 817–830. 10.1016/j.neuron.2006.09.040 17145503 PMC1899130

[B100] Parton R. G. Simons K. Dotti C. G. (1992). Axonal and dentritic endocytic pathways in cultured neurons. J. Cell Biol. 119, 123–137. 10.1083/jcb.119.1.123 1527164 PMC2289637

[B101] Passafaro M. Piëch V. Sheng M. (2001). Subunit-specific temporal and spatial patterns of AMPA receptor exocytosis in hippocampal neurons. Nat. Neurosci. 4, 917–926. 10.1038/nn0901-917 11528423

[B102] Patterson M. A. Szatmari E. M. Yasuda R. (2010). AMPA receptors are exocytosed in stimulated spines and adjacent dendrites in a Ras-ERK-dependent manner during long-term potentiation. Proc. Natl. Acad. Sci. U.S.A. 107, 15951–15956. 10.1073/pnas.0913875107 20733080 PMC2936631

[B103] Penn A. C. Zhang C. L. Georges F. Royer L. Breillat C. Hosy E.. (2017). Hippocampal LTP and contextual learning require surface diffusion of AMPA receptors. Nature 549, 384–388. 10.1038/nature23658 28902836 PMC5683353

[B104] Petersen C. C. Malenka R. C. Nicoll R. A. Hopfield J. J. (1998). All-or-none potentiation at CA3-CA1 synapses. Proc. Natl. Acad. Sci. U.S.A. 95, 4732–4737. 10.1073/pnas.95.8.4732 9539807 PMC22559

[B105] Petrini E. M. Lu J. Cognet L. Lounis B. Ehlers M. D. Choquet D. (2009). Endocytic trafficking and recycling maintain a pool of mobile surface AMPA receptors required for synaptic potentiation. Neuron 63, 92–105. 10.1016/j.neuron.2009.05.025 19607795 PMC2847611

[B106] Plant K. Pelkey K. A. Bortolotto Z. A. Morita D. Terashima A. McBain C. J.. (2006). Transient incorporation of native GluR2-lacking AMPA receptors during hippocampal long-term potentiation. Nat. Neurosci. 9, 602–604. 10.1038/nn1678 16582904

[B107] Pozo K. Cingolani L. A. Bassani S. Laurent F. Passafaro M. Goda Y. (2012). β3 integrin interacts directly with GluA2 AMPA receptor subunit and regulates AMPA receptor expression in hippocampal neurons. Proc. Natl. Acad. Sci. U.S.A. 109, 1323–1328. 10.1073/pnas.1113736109 22232691 PMC3268285

[B108] Rácz B. Blanpied T. A. Ehlers M. D. Weinberg R. J. (2004). Lateral organization of endocytic machinery in dendritic spines. Nat. Neurosci. 7, 917–918. 10.1038/nn1303 15322548

[B109] Rathje M. Fang H. Bachman J. L. Anggono V. Gether U. Huganir R. L.. (2013). AMPA receptor pHluorin-GluA2 reports NMDA receptor-induced intracellular acidification in hippocampal neurons. Proc. Natl. Acad. Sci. U.S.A. 110, 14426–14431. 10.1073/pnas.1312982110 23940334 PMC3761605

[B110] Roche K. W. Brien R. J. O. Mammen A. L. Bernhardt J. Huganir R. L. (1996). Characterization of multiple phosphorylation sites on the AMPA receptor GluR1 subunit. Neuron 16, 1179–1188. 10.1016/S0896-6273(00)80144-0 8663994

[B111] Roman-Vendrell C. Chevalier M. Acevedo-Canabal A. M. Delgado-Peraza F. Flores-Otero J. Yudowski G. A. (2014). Imaging of kiss-and-run exocytosis of surface receptors in neuronal cultures. Front. Cell. Neurosci. 8:363. 10.3389/fncel.2014.00363 25404895 PMC4217495

[B112] Saglietti L. Dequidt C. Kamieniarz K. Rousset M. Valnegri P. Thoumine O.. (2007). Extracellular Interactions between GluR2 and N-cadherin in spine regulation. Neuron 54, 461–477. 10.1016/j.neuron.2007.04.012 17481398

[B113] Schnell E. Sizemore M. Karimzadegan S. Chen L. Bredt D. S. Nicoll R. A. (2002). Direct interactions between PSD-95 and stargazin control synaptic AMPA receptor number. Proc. Natl. Acad. Sci. U.S.A. 99, 13902–13907. 10.1073/pnas.172511199 12359873 PMC129795

[B114] Schwenk J. Baehrens D. Haupt A. Bildl W. Boudkkazi S. Roeper J. (2014). Neuroresource regional diversity and developmental dynamics of the AMPA-receptor proteome in the mammalian brain. Neuron 84, 41–54. 10.1016/j.neuron.2014.08.044 25242221

[B115] Schwenk J. Harmel N. Brechet A. Zolles G. Berkefeld H. Müller C. S.. (2012). High-resolution proteomics unravel architecture and molecular diversity of native AMPA receptor complexes. Neuron 74, 621–633. 10.1016/j.neuron.2012.03.034 22632720

[B116] Shi S.-H. Hayashi Y. Petralia R. S. Zaman S. H. Wenthold R. J. Svoboda K.. (1999). Rapid spine delivery and redestribution of AMPA receptors after synaptic NMDA receptor activation. Science 284, 1811–1816. 10.1126/science.284.5421.1811 10364548

[B117] Sinnen B. L. Bowen A. B. Forte J. S. Hiester B. G. Crosby K. C. Gibson E. S.. (2017). Optogenetic control of synaptic composition and function. Neuron 93, 646–660. 10.1016/j.neuron.2016.12.037 28132827 PMC5300939

[B118] Soler-llavina G. J. Arstikaitis P. Morishita W. Ahmad M. Sudhof T. C. (2013). Report leucine-rich repeat transmembrane proteins are essential for maintenance of long-term potentiation. Neuron 79, 439–446. 10.1016/j.neuron.2013.06.007 23931994 PMC3741667

[B119] Son J. Keefe M. D. Stevenson T. J. Barrios J. P. Anjewierden S. Newton J. B.. (2016). Transgenic fingRs for live mapping of synaptic dynamics in genetically-defined neurons. Sci. Rep. 6:18734. 10.1038/srep18734 26728131 PMC4700522

[B120] Spacek J. Harris K. M. (1997). Three-dimensional organization of smooth endoplasmic reticulum in hippocampal CA1 dendrites and dendritic spines of the immature and mature Rat. J. Neurosci. 17, 190–203. 10.1523/JNEUROSCI.17-01-00190.1997 8987748 PMC6793680

[B121] Spence E. F. Kanak D. J. Carlson B. R. Soderling S. H. (2016). The Arp2/3 complex is essential for distinct stages of spine synapse maturation, including synapse unsilencing. J. Neurosci. 36, 9696–9709. 10.1523/JNEUROSCI.0876-16.2016 27629719 PMC5039249

[B122] Sun X. Zhao Y. Wolf M. E. (2005). Dopamine receptor stimulation modulates AMPA receptor synaptic insertion in prefrontal cortex neurons. J. Neurosci. 25, 7342–7351. 10.1523/JNEUROSCI.4603-04.2005 16093384 PMC6725299

[B123] Szepesi Z. Hosy E. Ruszczycki B. Bijata M. Pyskaty M. Bikbaev A.. (2014). Synaptically released matrix metalloproteinase activity in control of structural plasticity and the cell surface distribution of GluA1-AMPA receptors. PLoS ONE 9:e98274. 10.1371/journal.pone.0098274 24853857 PMC4031140

[B124] Szymczak A. L. Workman C. J. Wang Y. Vignali K. M. Dilioglou S. Vanin E. F.. (2004). Correction of multi-gene deficiency *in vivo* using a single ‘self-cleaving' 2A peptide-based retroviral vector. Nat. Biotechnol. 22, 589–595. 10.1038/nbt957 15064769

[B125] Tanaka J.-I. Horiike Y. Matsuzaki M. Miyazaki T. Ellis-Davies G. C. R. Kasai H. (2008). Protein synthesis and neurotrophin-dependent structural plasticity of single dendritic spines. Science 319, 1683–1687. 10.1126/science.1152864 18309046 PMC4218863

[B126] Tang A.-H. Chen H. Li T. P. Metzbower S. R. MacGillavry H. D. Blanpied T. A. (2016). A trans-synaptic nanocolumn aligns neurotransmitter release to receptors. Nature 536, 210–214. 10.1038/nature19058 27462810 PMC5002394

[B127] Tao-Cheng J.-H. Crocker V. T. Winters C. A. Azzam R. Chludzinski J. Reese T. S. (2011). Trafficking of AMPA receptors at plasma membranes of hippocampal neurons. J. Neurosci. 31, 4834–4843. 10.1523/JNEUROSCI.4745-10.2011 21451021 PMC3138201

[B128] Tardin C. Cognet L. Bats C. Lounis B. Choquet D. (2003). Direct imaging of lateral movements of AMPA receptors inside synapses. EMBO J. 22, 4656–4665. 10.1093/emboj/cdg463 12970178 PMC212729

[B129] Tomita S. Chen L. Kawasaki Y. Petralia R. S. Wenthold R. J. Nicoll R. A.. (2003). Functional studies and distribution define a family of transmembrane AMPA receptor regulatory proteins. J. Cell Biol. 161, 805–816. 10.1083/jcb.200212116 12771129 PMC2199354

[B130] Tomita S. Fukata M. Nicoll R. Bredt D. (2004). Dynamic interaction of stargazing-like TARPs with cycling AMPA receptors at synapses. Science 303, 1508–1511. 10.1126/science.1090262 15001777

[B131] Traynelis S. F. Wollmuth L. P. McBain C. J. Menniti F. S. Vance K. M. Ogden K. K.. (2010). Glutamate receptor ion channels: structure, regulation, and function. Pharmacol. Rev. 62, 405–496. 10.1124/pr.109.002451 20716669 PMC2964903

[B132] Uezu A. Kanak D. J. Bradshaw T. W. A. Soderblum E. J. Catavero C. M. Burette A. C.. (2016). Identification of an elaborate complex mediating postsynaptic inhibition. Science 353, 960–962. 10.1126/science.aag0821 27609886 PMC5432043

[B133] Wakayama S. Kiyonaka S. Arai I. Kakegawa W. Matsuda S. Ibata K.. (2017). Chemical labelling for visualizing native AMPA receptors in live neurons. Nat. Commun. 8:14850. 10.1038/ncomms14850 28387242 PMC5385570

[B134] Walker A. S. Neves G. Grillo F. Jackson R. E. Rigby M. Donnell C. O.. (2017). Distance-dependent gradient in NMDAR-driven spine calcium signals along tapering dendrites. Proc. Natl. Acad. Sci. U.S.A. 114, E1986–E1995. 10.1073/pnas.1607462114 28209776 PMC5347575

[B135] Wang R. Walker C. S. Brockie P. J. Francis M. M. Mellem J. E. Madsen D. M.. (2008a). Evolutionary conserved role for TARPs in the gating of glutamate receptors and tuning of synaptic function. Neuron 59, 997–1008. 10.1016/j.neuron.2008.07.023 18817737 PMC2754846

[B136] Wang X. Bozdagi O. Nikitczuk J. S. Zhai Z. W. Zhou Q. Huntley G. W. (2008b). Extracellular proteolysis by matrix metalloproteinase-9 drives dendritic spine enlargement and long-term potentiation coordinately. Proc. Natl. Acad. Sci. U.S.A. 105, 19520–19525. 10.1073/pnas.0807248105 19047646 PMC2614793

[B137] Wang Z. Edwards J. G. Riley N. Provance D. W. Karcher R. Li X.-D.. (2008c). Myosin Vb mobilizes recycling endosomes and AMPA receptors for postsynaptic plasticity. Cell 135, 535–548. 10.1016/j.cell.2008.09.057 18984164 PMC2585749

[B138] Watson J. F. Ho H. Greger I. H. (2017). Synaptic transmission and plasticity require AMPA receptor anchoring via its N-terminal domain. Elife 6:e23024. 10.7554/eLife.23024 28290985 PMC5370185

[B139] Werner C. T. Murray C. H. Reimers J. M. Chauhan N. M. Woo K. K. Y. Molla H. M.. (2017). Trafficking of calcium-permeable and calcium-impermeable AMPA receptors in nucleus accumbens medium spiny neurons co-cultured with prefrontal cortex neurons. Neuropharmacology 116, 224–232. 10.1016/j.neuropharm.2016.12.014 27993521 PMC5385156

[B140] Woolfrey K. M. Sanderson J. L. Dell'Acqua M. L. (2015). The palmitoyl acyltransferase DHHC2 regulates recycling endosome exocytosis and synaptic potentiation through palmitoylation of AKAP79/150. J. Neurosci. 35, 442–456. 10.1523/JNEUROSCI.2243-14.2015 25589740 PMC4293401

[B141] Wu D. Bacaj T. Morishita W. Goswami D. Arendt K. L. Xu W.. (2017a). Postsynaptic synaptotagmins mediate AMPA receptor exocytosis during LTP. Nature 544, 316–321. 10.1038/nature21720 28355182 PMC5734942

[B142] Wu Y. Whiteus C. Xu C. S. Hayworth K. J. Weinberg R. J. Hess H. F. (2017b). Contacts between the endoplasmic reticulum and other membranes in neurons. *Proc. Natl. Acad. Sci*. U.S.A. 114, E4859–E4867. 10.1073/pnas.1701078114 PMC5474793 28559323

[B143] Yang Y. Wang X. Frerking M. Zhou Q. (2008b). Spine expansion and stabilization associated with long-term potentiation. J. Neurosci. 28, 5740–5751. 10.1523/JNEUROSCI.3998-07.2008 18509035 PMC2561912

[B144] Yang Y. Wang X.-B. Frerking M. Zhou Q. (2008a). Delivery of AMPA receptors to perisynaptic sites precedes the full expression of long-term potentiation. Proc. Natl. Acad. Sci. U.S.A. 105, 11388–11393. 10.1073/pnas.0802978105 18682558 PMC2496888

[B145] Ye T. Ip J. P. K. Fu A. K. Y. Ip N. Y. (2014). Cdk5-mediated phosphorylation of RapGEF2 controls neuronal migration in the developing cerebral cortex. Nat. Commun. 5:4826. 10.1038/ncomms5826 25189171 PMC4164783

[B146] Yudowski G. A. Puthenveedu M. A. Leonoudakis D. Panicker S. Thorn K. S. Beattie E. C.. (2007). Real-time imaging of discrete exocytic events mediating surface delivery of AMPA receptors. J. Neurosci. 27, 11112–11121. 10.1523/JNEUROSCI.2465-07.2007 17928453 PMC3249441

[B147] Zhang Y. Cudmore R. H. Lin D. Linden D. J. Huganir R. L. (2015). Visualization of NMDA receptor–dependent AMPA receptor synaptic plasticity *in vivo*. Nat. Neurosci. 18, 402–407. 10.1038/nn.3936 25643295 PMC4339371

[B148] Zheng N. Jeyifous O. Munro C. Montgomery J. M. Green W. N. (2015). Synaptic activity regulates AMPA receptor trafficking through different recycling pathways. Elife 4:e06878. 10.7554/eLife.06878 25970033 PMC4451724

