# Supplementary material for: Mechanisms and Role of Dendritic Membrane Trafficking for Long-Term Potentiation
Source: Front Cell Neurosci. 2018 Oct 30;12:391. doi: 10.3389/fncel.2018.00391 (PMC6218485; doi:10.3389/fncel.2018.00391)
Supplement: Supplementary Figure 1 — ER acidification suppresses SEP-GluA1 signal during cLTP. (A) Method for masking the dendritic shaft for SEP-GluA1 signal analysis. A mask was generated so that the dendritic shaft signal could be selectively quantified. This was generated by subtracting a synapse mask, based on the SEP-GluA1 signal from a whole cell mask generated from the mCh cell fill (see methods below for details). Shown is the mCh cell fill (panel i), binary synapse mask based on the SEP-GluA1 signal (red, panel ii) and the SEP-GluA1 signal (gray scale, panel iii) with dendritic shaft mask overlayed (burgundy). Scale bar 10 μm. (B) Quantification of the spine (solid line) and shaft (dashed line) SEP-GluA1 signal as a function of time during and after cLTP (gray bar). Note the robust decrease in shaft signal, which slightly precedes synaptic accumulation. Error bars represent standard error of the mean.This decrease is likely due to ER acidification as outlined in Rathje et al. (2013). [file Presentation_1.pdf]

Supplementary Figure 1

A

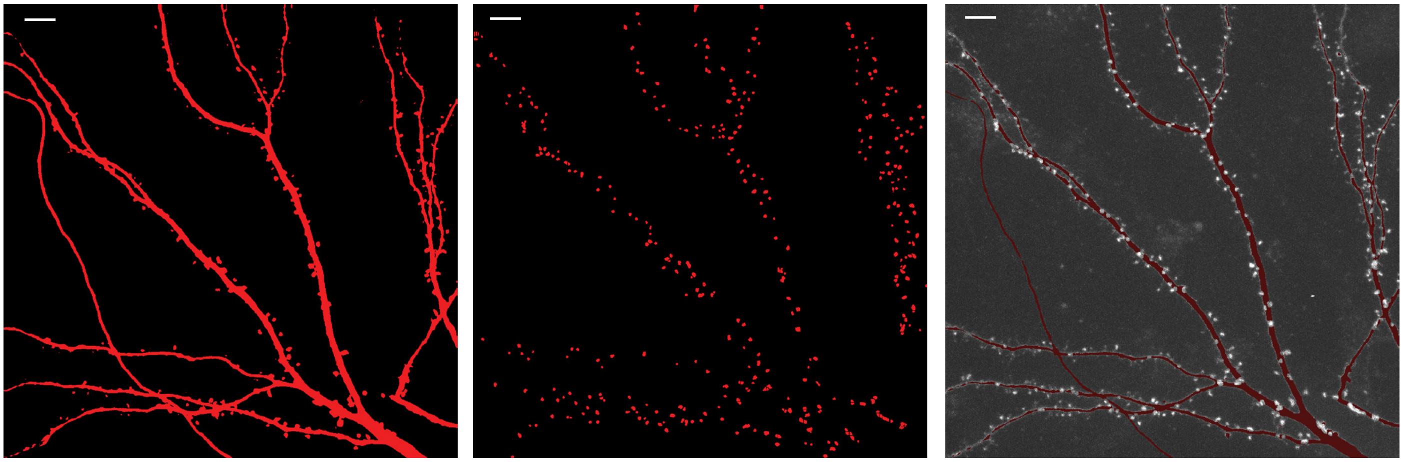

B

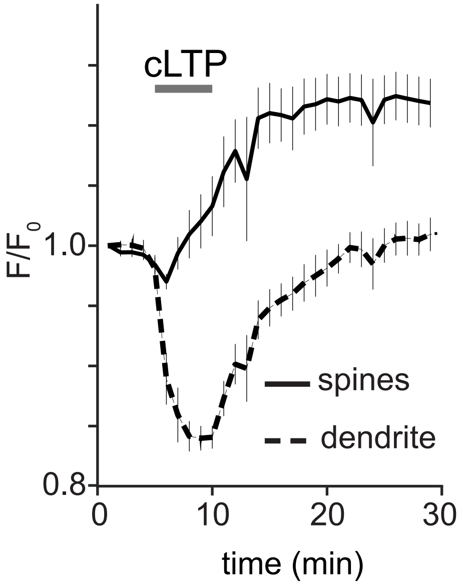

Supplementary Figure 1: ER acidification suppresses SEP-GluA1 signal during cLTP

A. Method for masking the dendritic shaft for SEP-GluA1 signal analysis: A dendritic shaft mask was generated to selectively quantify dendritic shaft signal by subtracting a synapse mask based on SEP-GluA1 signal from a mask of the whole cell (see methods below for details). Shown is the whole cell mask based on mCh cell fill signal (panel i), the synapse mask based on the SEP-GluA1 signal (red, panel ii) and the fluorescent SEP-GluA1 signal (grey scale, panel iii) with resulting dendritic shaft mask overlayed (burgundy). Scale bar 10 µm
B. Quantification of the spine and shaft SEP-GluA1 signal as a function of time during and after cLTP. Note the robust decrease in shaft signal, which slightly precedes synaptic accumulation. Error bars represent standard error of the mean. This decrease is likely due to ER acidification as outlined in Rathje et al. (PNAS, 2013; 110:14426).

Methods for selectively analyzing dendritic shaft SEP-GluA1 signal:
The intensity within the dendritic shaft was identified and quantified using a custom image filtering and masking routine implemented in Matlab along with the freely available Matlab package DipImage (Delft University of Technology). To correct for any illumination non-uniformity, all images were first tophat filtered; the morphological opening of the image (computed using the DipImage 'opening' function with a 5 pixel 'elliptic' structuring element) was subtracted from the original image. To identify SEP-GluA1 accumulation at spines (Supplementary Fig. 1A, panel ii), regions of high intensity within the image were identified using a set of two sequential image filters; a second derivative filter in the gradient dimension was implemented using the DipImage function 'dgg' followed by a variance filter with a 5 pixel 'elliptic' structuring element implemented using the DipImage fuction 'varif'. The resulting filtered image was then automatically thresholded using the DipImage 'threshold' function. A corresponding whole cell mask was also generated for each image using the mCh cell fill signal (Supplementary Fig. 1A, panel i). Images were first smoothed using a gaussian filter with sigma = 1, followed by automatic thresholding implemented using the DipImage 'gaussf' and 'threshold' functions respectively. The masked SEP-GluA1 regions were then removed from the whole cell mask to generate a final mask representing only the dendritic shaft areas (Supplementary Fig. 1A, panel iii). The intensity within this region was then used for quantification of the dendrite intensity.
